# Supplementary figures and images for: Insomnia and risk of all-cause dementia: A systematic review and meta-analysis
Source: PLoS One. 2025 Apr 9;20(4):e0318814. doi: 10.1371/journal.pone.0318814 (PMC11981150; doi:10.1371/journal.pone.0318814)

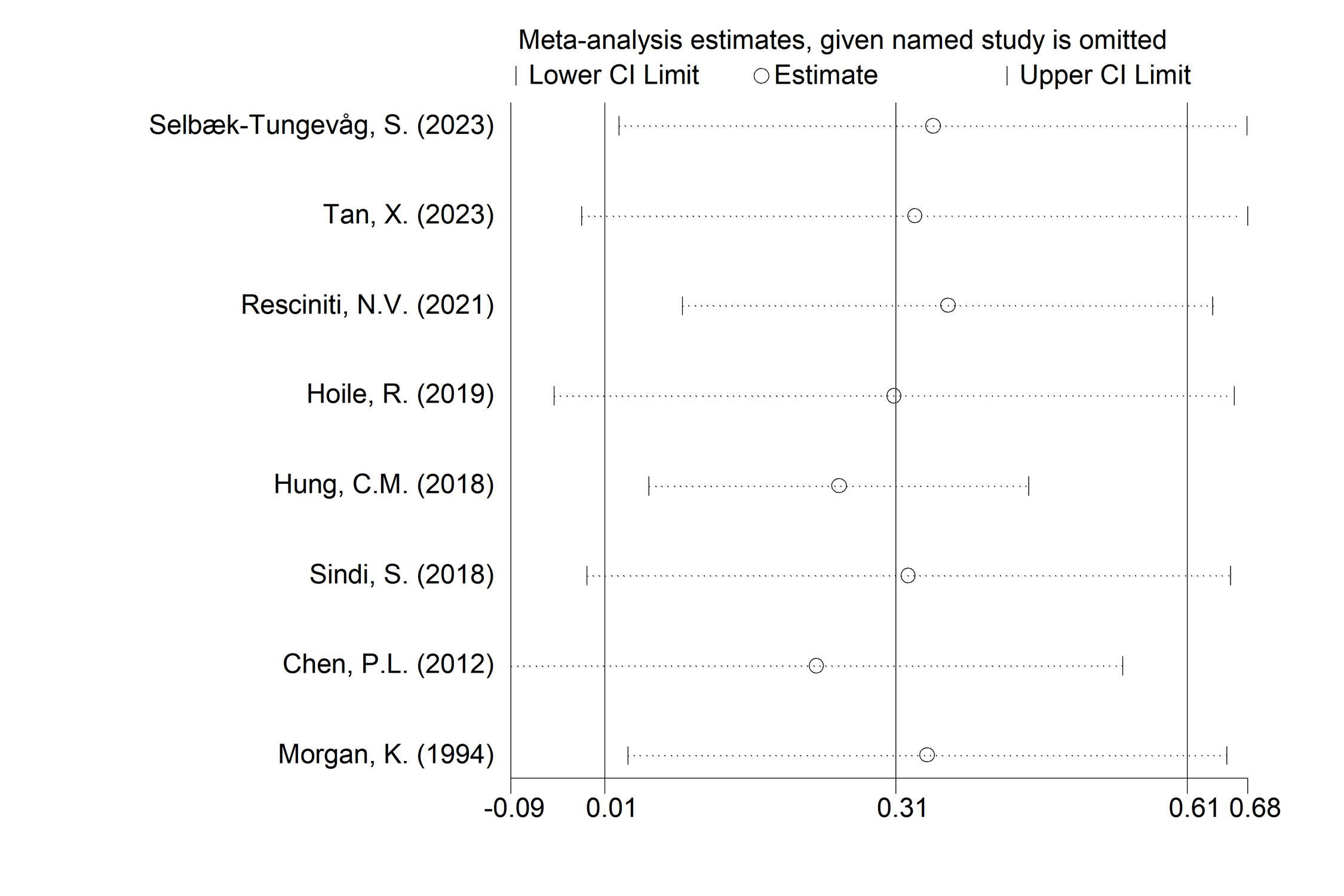

Supplement: S1 Fig — (TIF) [file pone.0318814.s001.tif]

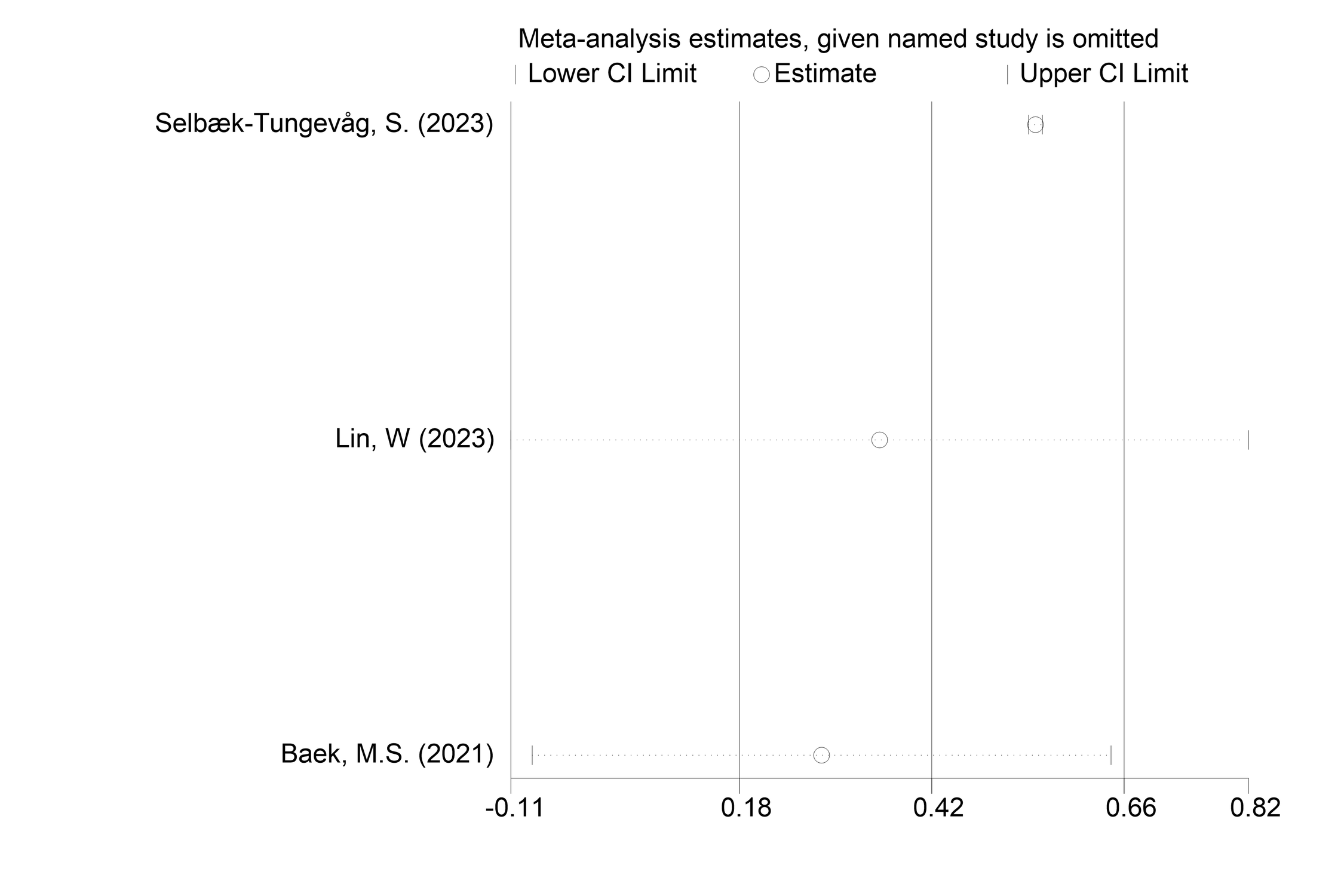

Supplement: S2 Fig — (TIF) [file pone.0318814.s002.tif]

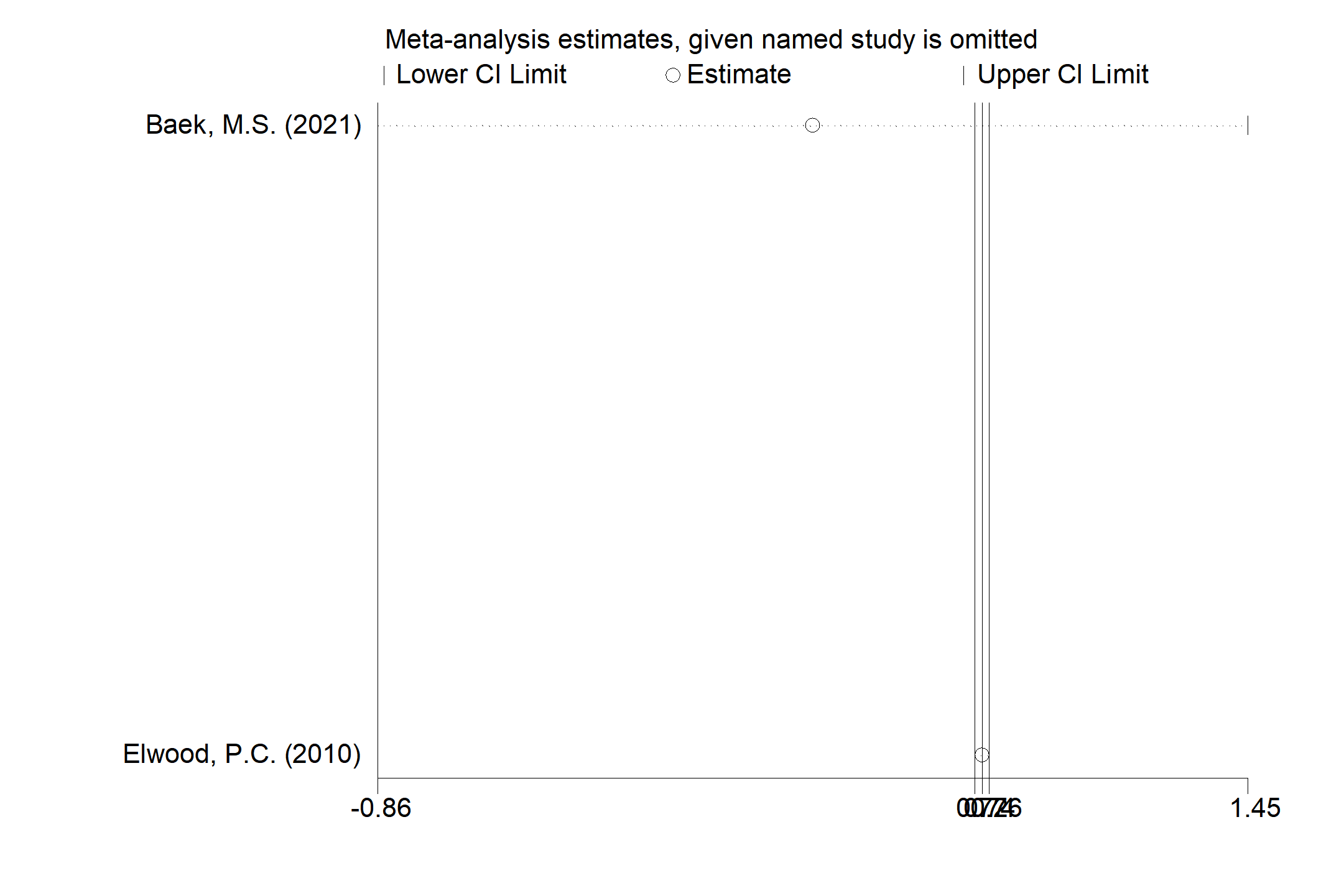

Supplement: S3 Fig — (TIF) [file pone.0318814.s003.tif]

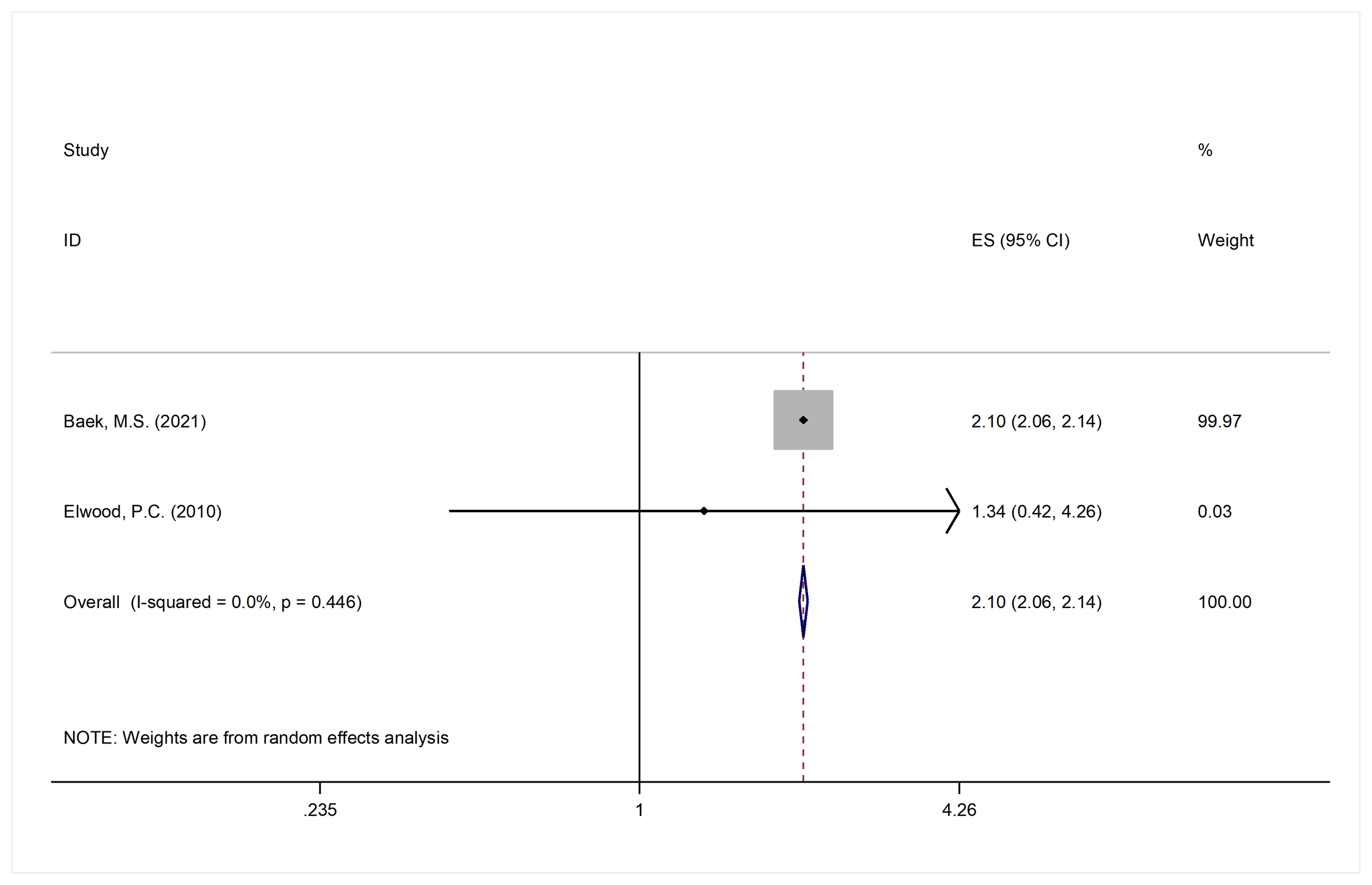

Supplement: S4 Fig — (TIF) [file pone.0318814.s004.tif]

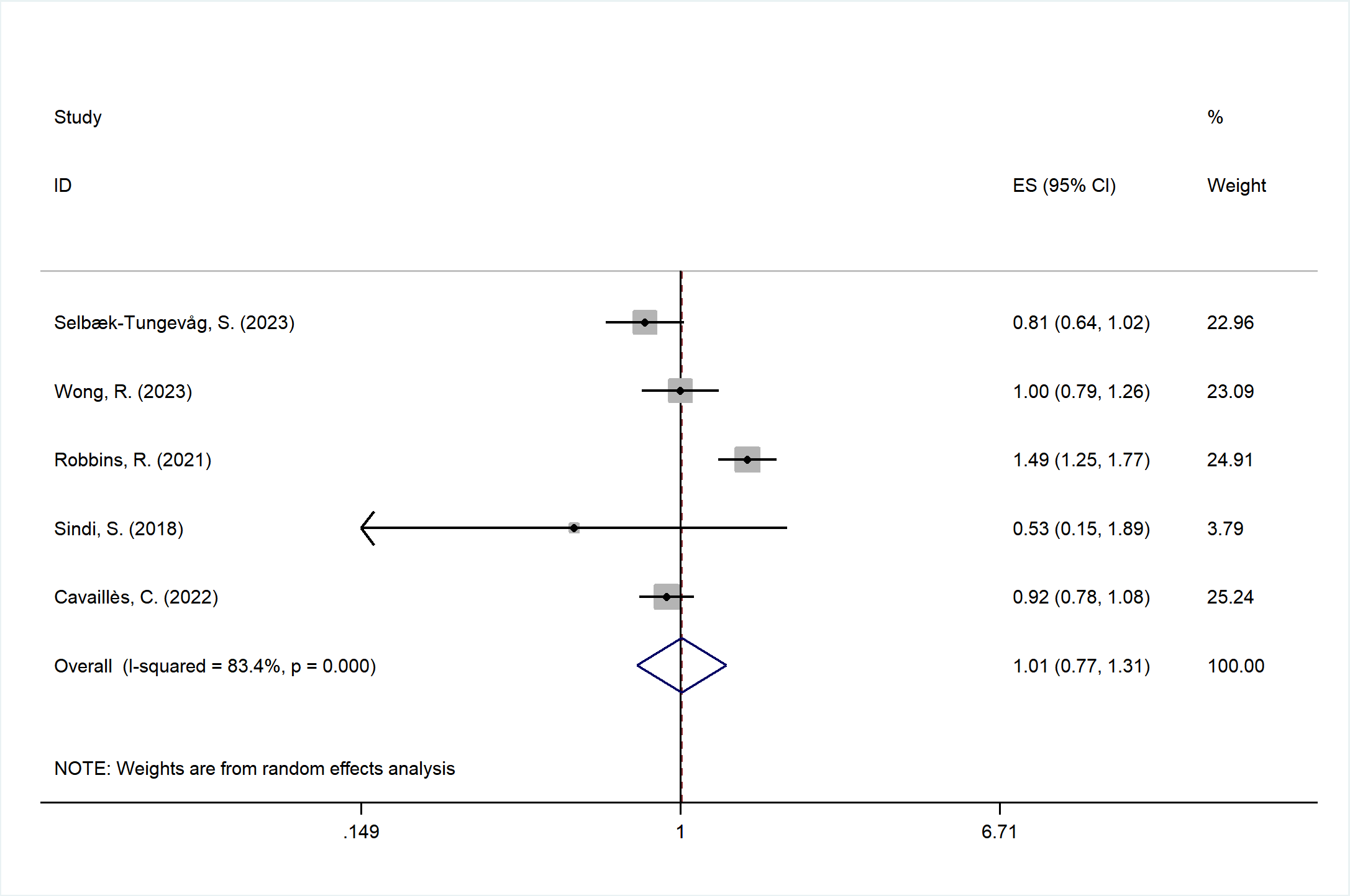

Supplement: S5 Fig — (TIF) [file pone.0318814.s005.tif]

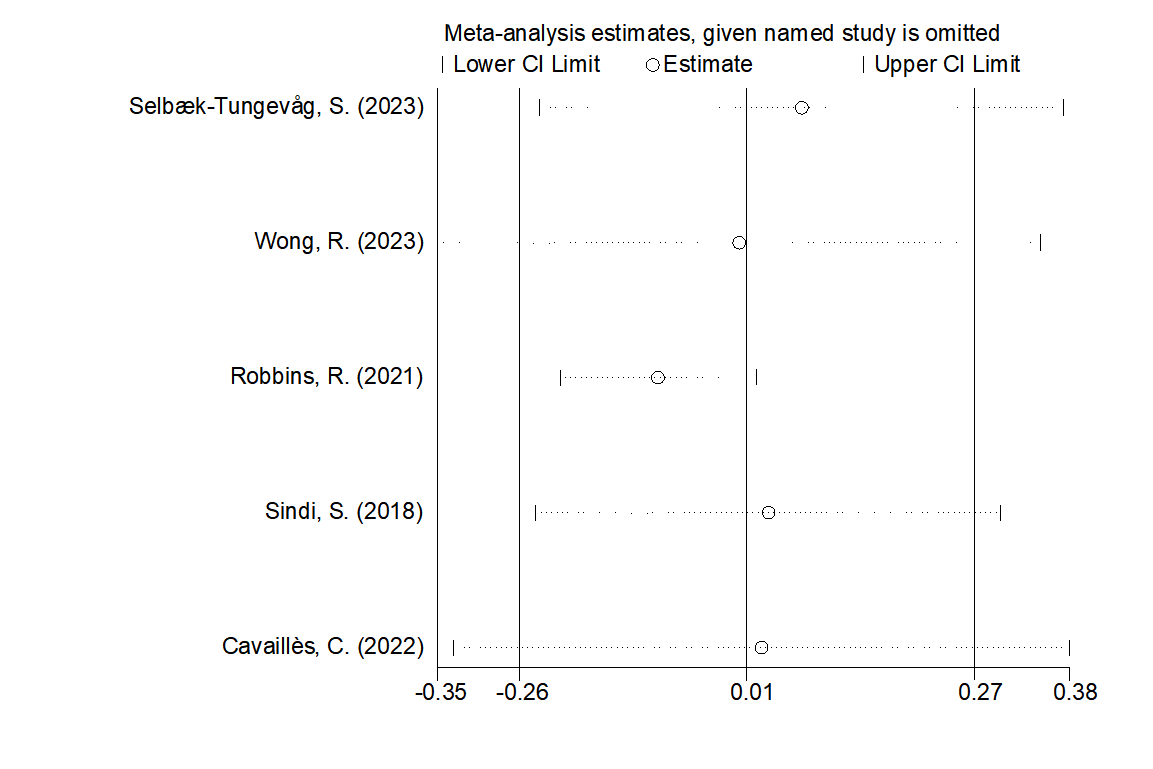

Supplement: S6 Fig — (TIF) [file pone.0318814.s006.tif]

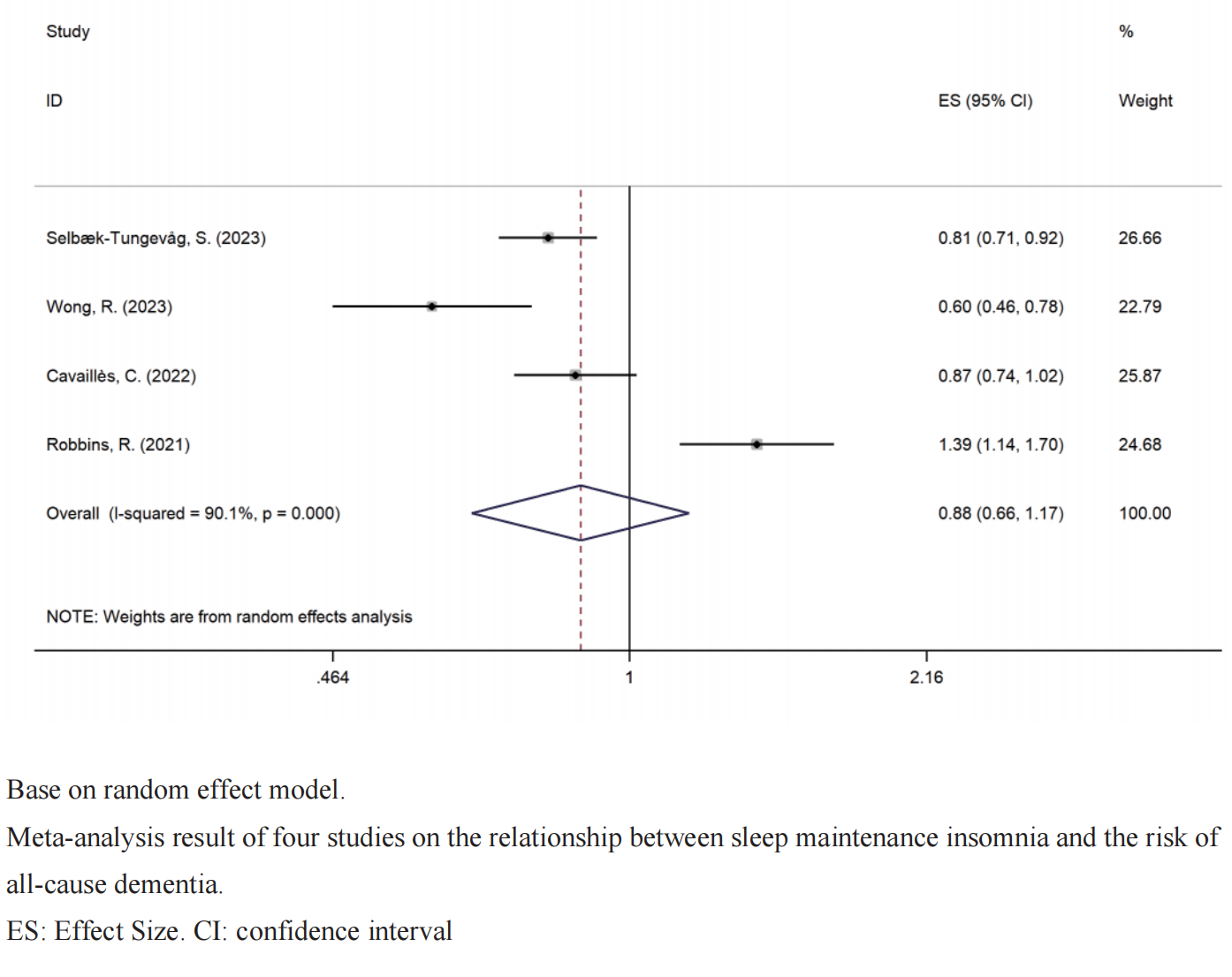

Supplement: S7 Fig — (TIF) [file pone.0318814.s007.tif]

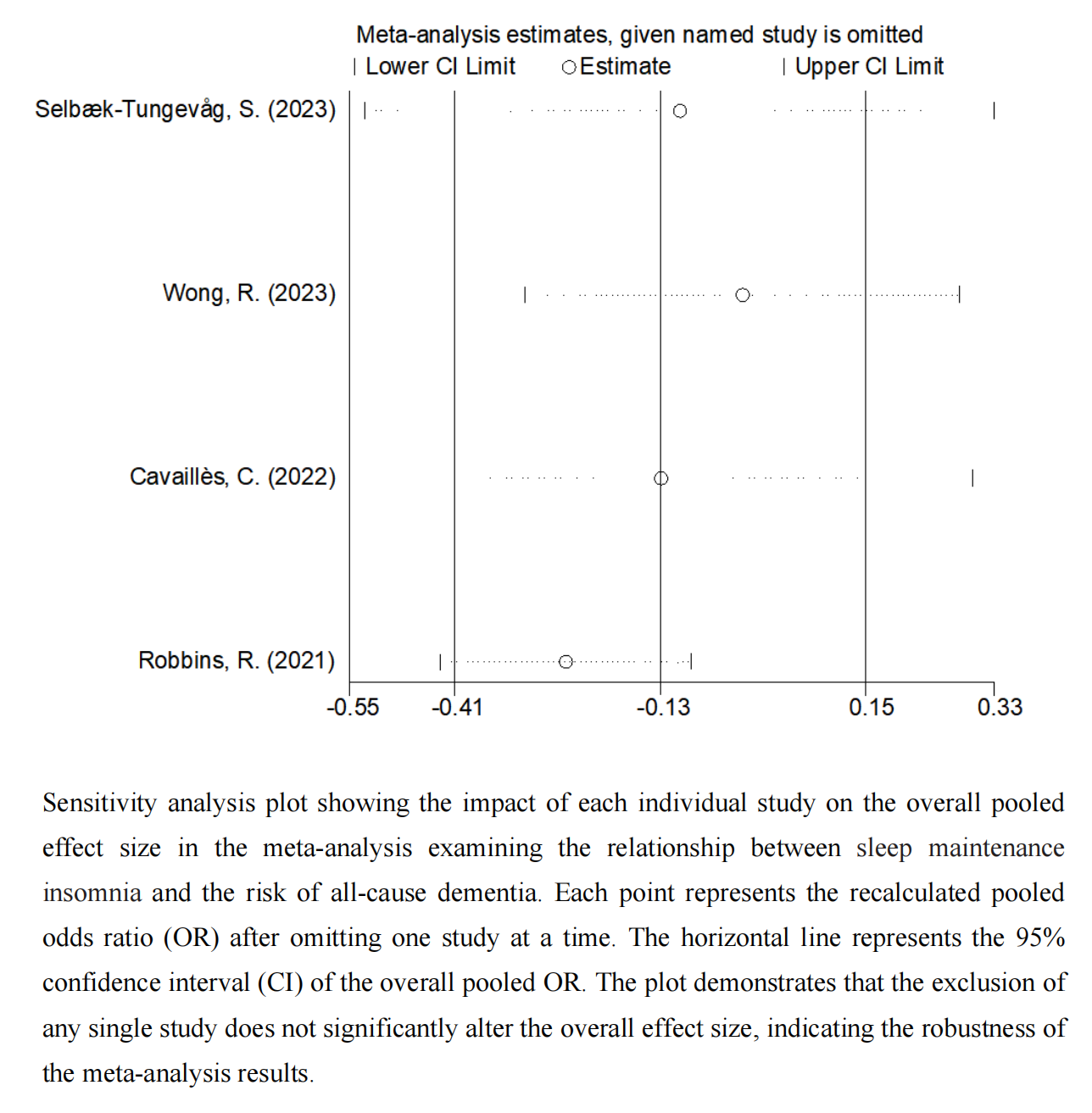

Supplement: S8 Fig — (TIF) [file pone.0318814.s008.tif]

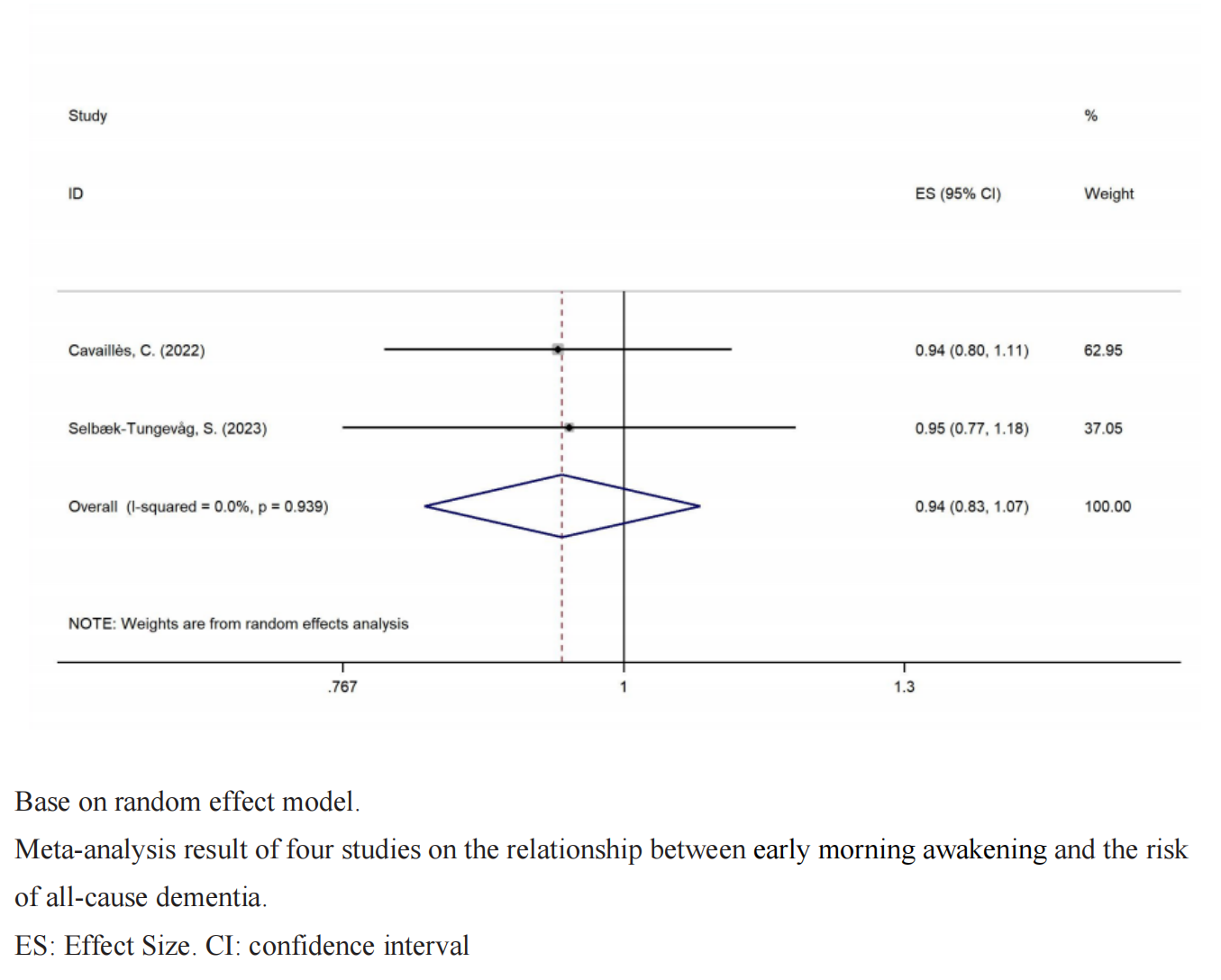

Supplement: S9 Fig — (TIF) [file pone.0318814.s009.tif]

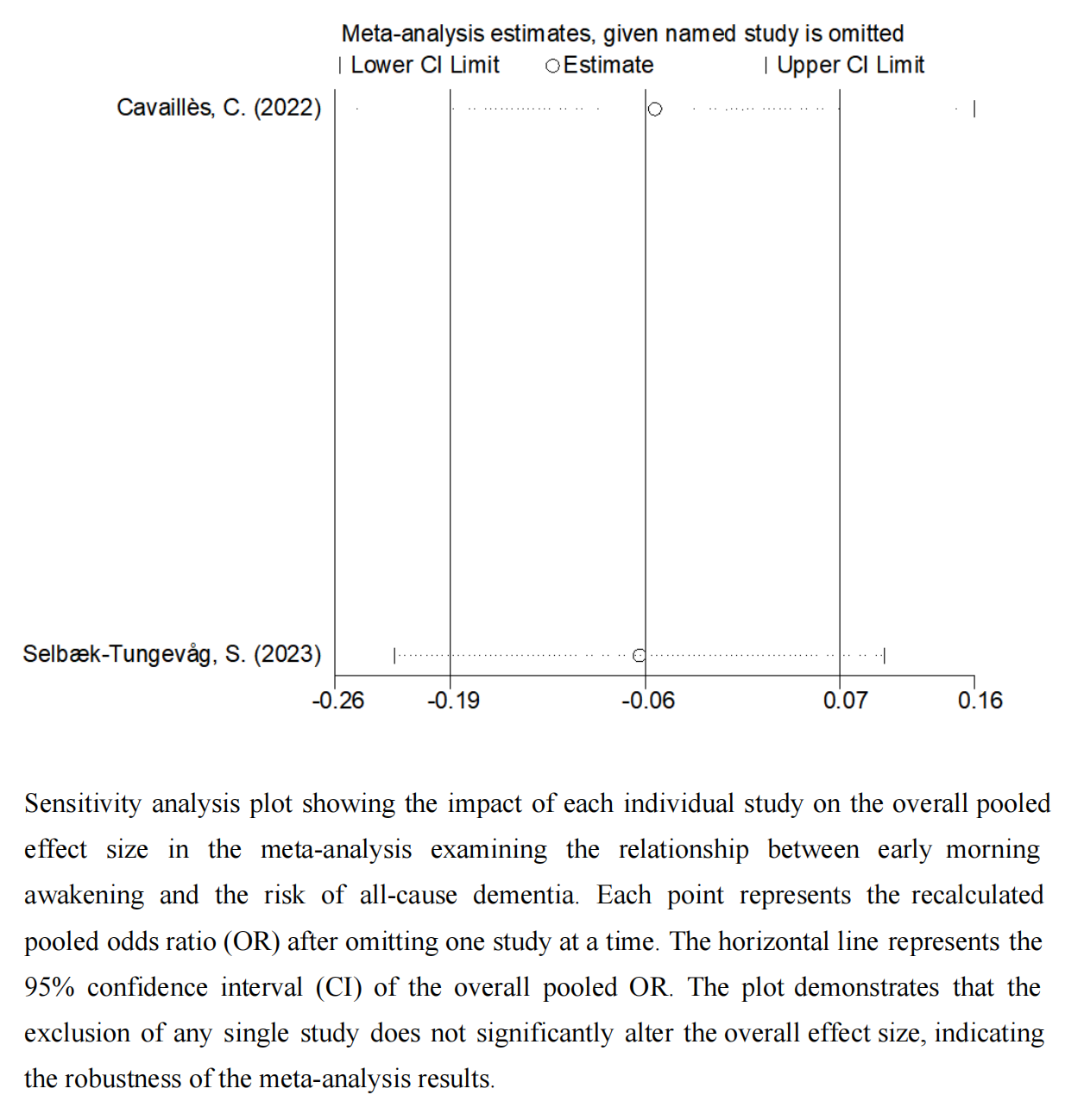

Supplement: S10 Fig — (TIF) [file pone.0318814.s010.tif]
