# Supplementary material for: Insomnia and risk of all-cause dementia: A systematic review and meta-analysis
Source: PLoS One. 2025 Apr 9;20(4):e0318814. doi: 10.1371/journal.pone.0318814 (PMC11981150; doi:10.1371/journal.pone.0318814)
Supplement: S1 File — Supplementary Table S1-S3 Detailed search strategies. Table S1: PubMed. Table S2: Embase. Table S3: Cochran Library. (DOC) [file pone.0318814.s011.doc]

**The retrieval strategies and retrieval results of each database are shown in Tables 1-3**.

Supplementary Table 1: PubMed

| No. | Content | Result |
| --- | --- | --- |
| #1 | Search: Sleep Initiation and Maintenance Disorders[MeSH]  Sort by: Most Recent | 18,365 |
| #2 | Search: (((((Disorders of Initiating[Title/Abstract] AND Maintaining Sleep[Title/Abstract]) OR (DIMS[Title/Abstract])) OR (Early Awakening[Title/Abstract])) OR (Insomnia*[Title/Abstract])) OR (Sleep Initiation Dysfunction*[Title/Abstract])) OR (Sleeplessness[Title/Abstract]) Sort by: Most Recent | 32,342 |
| #3 | #1 OR #2 | 37,317 |
| #4 | Search: (Dementia[MeSH]) OR (Alzheimer Disease[MeSH])  Sort by: Most Recent | 210,363 |
| #5 | Search: ((((Dementia*[Title/Abstract]) OR (Amentia*[Title/Abstract])) OR (Alzheimer Dementia*[Title/Abstract])) OR (Senile Dementia*[Title/Abstract])) OR (Alzheimer*[Title/Abstract]) Sort by: Most Recent | 287,076 |
| #6 | #4 OR #5 | 329,513 |
| #7 | Search: Risk[MeSH] Sort by: Most Recent | 1,398,014 |
| #8 | Search: Risk*[Title/Abstract] Sort by: Most Recent | 3,018,717 |
| #9 | Search: (Risk[MeSH]) OR (Risk*[Title/Abstract])  Sort by: Most Recent | 3,513,679 |
| #10 | #3 AND #6 AND #9 | 409 |

Supplementary Table 2: Embase

| No. | Content | Result |
| --- | --- | --- |
| #1 | 'insomnia'/exp | 90,196 |
| #2 | ((((('disorders of initiating':ti,ab,kw AND 'maintaining sleep':ti,ab,kw) OR ('dims':ti,ab,kw)) OR ('early awakening':ti,ab,kw)) OR ('insomnia*':ti,ab,kw)) OR ('sleep initiation dysfunction*':ti,ab,kw)) OR ('sleeplessness':ti,ab,kw) | 54,019 |
| #3 | #1 OR #2 | 100,507 |
| #4 | ('dementia'/exp) OR ('Alzheimer disease'/exp) | 457,506 |
| #5 | (((('dementia*':ti,ab,kw) OR ('amentia*':ti,ab,kw)) OR ('alzheimer dementia*':ti,ab,kw)) OR ('senile dementia*':ti,ab,kw)) OR ('alzheimer*':ti,ab,kw) | 397,901 |
| #6 | #4 OR #5 | 533,759 |
| #7 | 'risk'/exp | 3,165,750 |
| #8 | 'risk*':ti,ab,kw | 4,356,640 |
| #9 | #7 OR #8 | 5,203,540 |
| #10 | #3 AND #6 AND #9 | 1476 |

Supplementary Table 3: Cochran Library

| No. | Content | Result |
| --- | --- | --- |
| #1 | MeSH descriptor: [Sleep Initiation and Maintenance Disorders] explode all trees | 3,384 |
| #2 | (Disorders of Initiating OR Maintaining Sleep OR DIMS OR Early Awakening OR Insomnia* OR Sleep Initiation Dysfunction* OR Sleeplessness):ti,ab,kw | 16,015 |
| #3 | #1 OR #2 | 17,635 |
| #4 | MeSH descriptor: [Parkinson Disease] explode all trees | 9395 |
| #5 | MeSH descriptor: [Alzheimer Disease] explode all trees | 5429 |
| #6 | (Dementia* OR Amentia* OR Alzheimer Dementia* OR Senile Dementia* OR Alzheimer*):ti,ab,kw | 24,945 |
| #7 | #4 OR #5 OR #6 | 12,508 |
| #8 | MeSH descriptor: [Risk] explode all trees | 58,597 |
| #9 | (Risk*):ti,ab,kw | 304,395 |
| #10 | #8 OR #9 | 308,094 |
| #11 | #3 AND #7 AND #10 | 99 |
