# Supplementary material for: Insomnia and risk of all-cause dementia: A systematic review and meta-analysis
Source: PLoS One. 2025 Apr 9;20(4):e0318814. doi: 10.1371/journal.pone.0318814 (PMC11981150; doi:10.1371/journal.pone.0318814)
Supplement: S5 Table — (DOC) [file pone.0318814.s013.doc]

Supplementary Table S5 | Reasons for excluded studies

| **Number** | **Study** | **Title** | **Authors** | **DIO** | **Exclusion reason** |
| --- | --- | --- | --- | --- | --- |
| 1 | Huang, C. H.2023 | Acupuncture is associated with reduced dementia risk in patients with insomnia: A propensity-score-matched cohort study of real-world data | Huang, C H;Lin, S K;Lin, M C;Hsieh, C L | 10.1016/j.jtcme.2023.02.003 | Not consistent with the theme |
| 2 | Dzierzewski, J. M.2020 | Cognitive functioning before and after insomnia treatment in women veterans | Dzierzewski, J M;  Zhu, R;Donovan, E K;  Perez, E;Song, Y;  Kelly, M R;Carlson, G;Fung, C H;Alessi, C;Martin, J L | 10.1093/sleep/zsaa056.534 | Not consistent with the theme |
| 3 | Ling, T. C.2022 | Development and validation of the dialysis dementia risk score: A retrospective, population-based, nested case-control study | Ling, T C; Chang, C C; Li, C Y; Sung, J M; Sun, C Y;Tsai, K J;Cheng, Y Y;Wu, J L;Kuo, Y T;Chang, Y T | 10.1111/ene.15123 | Not consistent with the theme |
| 4 | Lin, H.2016 | Factors associated with 90-day or 180-day long-term use of anxiety-hypnotic prescriptions and economic and safety outcomes: A retrospective cohort study | Lin, H; Tsai, H;Liu, F; Liao, C;Li, C; Lin, C; Chou, C; Chang, C; Simonavice, C A; Li, T |  | Not consistent with the theme |
| 5 | Li, G.2022 | Identification of medical conditions as risk factors for mild cognitive impairment - a US claims database study | Li, G; Nicola, T; Richard, B;  James, G; David, H;  Susan, D S; Harald, H | 10.14283/jpad.2022.97 | Not consistent with the theme |
| 6 | Camargos, E F.2011 | Incidence of sleep disorders in patients with Alzheimer disease | Camargos, E F;Pandolfi, M B  Dias, M P;Quintas, J L  Guimarães, R M; Nóbrega, Ode T | 10.1590/S1679-45082011AO2145 | Not consistent with the theme |
| 7 | Co, O.2019 | Insomnia increases risk of future development of cognitive impairments in patients with type 2 diabetes: a prospective study | Co, O; Siu, B W H; Ding, C  Luk, A O Y; Chow, E Y K  Ma, R C W;Chan, J C N  Kong, A P S | 10.1007/s00125-019-4946-6 | Not consistent with the theme |
| 8 | Diem, S. J.2016 | Measures of Sleep-Wake Patterns and Risk of Mild Cognitive Impairment or Dementia in Older Women | Diem, S J; Blackwell, T L  Stone, K L; Yaffe, K  Tranah, G; Cauley, J A  Ancoli-Israel, S; Redline, S; Spira, A P; Hillier, T A;Ensrud, K E | 10.1016/j.jagp.2015.12.002 | Not consistent with the theme |
| 9 | Aakre, J.A.2023 | Self-reported sleep fragmentation and sleep duration and their association with cognitive function in PROTECT, a large digital community-based cohort of people over 50 | Aakre, J A; Schulz, J; Ballard, C; Corbett, A; Bjorvatn, B; Aarsland, D; Creese, B; Hampshire, A; Brooker, H; Testad, I | 10.1002/gps.6022 | Not consistent with the theme |
| 10 | Fernandez-Mendoza, J.2016 | Short sleep duration modifies the relationship between cognitive impairment associated with cardiovascular disease and all-cause mortality | Fernandez-Mendoza, J  He, F; Vgontzas, A N; Liao, D; Bixler, E O |  | Not consistent with the theme |
| 11 | Ramakers, I H.2010 | Affective symptoms as predictors of Alzheimer's disease in subjects with mild cognitive impairment: a 10-year follow-up study. | Ramakers, I H;Visser, P J  Aalten, P;Kester, A  Jolles, J;Verhey, F R | 10.1017/S0033291709991577 | Without interest outcomes |
| 12 | Choe, Y.M.2022 | Association of a History of Sleep Disorder With Risk of Mild Cognitive Impairment and Alzheimer’s Disease Dementia. | Choe, Y. M.; Suh, G. H.; Kim, J. W. | 10.30773/pi.2022.0176 | Without interest outcomes |
| 13 | Jirong, Y.2013 | Association of sleep quality and dementia among long-lived Chinese older adults. | Jirong, Y; Changquan, H  Hongmei, W; Bi-Rong, D | 10.1007/s11357-012-9432-8 | Without interest outcomes |
| 14 | Mookerjee, N.2023 | Comorbidities and Risk Factors Associated With Insomnia in the Elderly Population | Mookerjee, N; Schmalbach, N;Antinori, G; Thampi, S; Windle-Puente, D; Gilligan, A; Huy, H  Andrews, M; Sun, A  Gandhi, R; Benedict, W  Chang, A; Sanders, B  Nguyen, J; Keesara, M R  Aliev, J; Patel, A  Hughes, I; Millstein, I  Hunter, K; Roy, S | 10.1177/21501319231168721 | Without interest outcomes |
| 15 | Merlino, G.2010 | Daytime sleepiness is associated with dementia and cognitive decline in older Italian adults: A population-based study | Merlino, G; Piani, A  Gigli, G L; Cancelli, I  Rinaldi, A; Baroselli, A  Serafini, A; Zanchettin, B  Valente, M | 10.1016/j.sleep.2009.07.018 | Without interest outcomes |
| 16 | Johar, H.2016 | Impaired Sleep Predicts Cognitive Decline in Old People: Findings from the Prospective KORA Age Study | Johar, H; Kawan, R  Emeny, R T; Ladwig, K H | 10.5665/sleep.5352 | Without interest outcomes |
| 17 | Sung, P S.2017 | Increased risk of dementia in patients with non-apnea sleep disorder | Sung, P S; Yeh, C C  Wang, L C; Hung, P H  Muo, C H; Sung, F C  Chen, C H; Tsai, K J | 10.2174/1567205013666161108104703 | Without interest outcomes |
| 18 | Beydoun, H. A.2021 | Insomnia as a predictor of diagnosed memory problems: 2006–2016 Health and Retirement Study | Beydoun, H A;Beydoun, M A;Weiss, J;Hossain, S  Huang, S;Alemu, B T  Zonderman, A B | 10.1016/j.sleep.2021.01.038 | Without interest outcomes |
| 19 | Basta, M.2016 |  | Basta, M;Koutentaki, E  Bertsias, A;Zaganas, I  Duijker, G;Panagiotakis, S;Lionis, C;Simos, P  Vgontzas, A | 10.1111/jsr.12446 | Without interest outcomes |
| 20 | Lai, V.2022 | Is sleep quality a potential predictor of neurocognitive disorders? A 6-year follow-up study in Chinese older adults | Lai, VKY;Fung, AWT  Lam, LCW;Lee, ATC | 10.1002/gps.5783 | Without interest outcomes |
| 21 | Guarnieri, B.2010 | Italian multicentric study on sleep disorders in mild cognitive impairment and dementias. | Guarnieri, B;Musicco, M;Appollonio, I  Caffarra, P;Bonanni, E  Ferri, R;Lombardi, G  Mearelli, S;Nobili, F  Perri, R;Rocchi, R  Sorbi, S | 10.1111/j.1365-2869.2010.00868.x | Without interest outcomes |
| 22 | Xie, B.2021 | Prevalence and risk factors of the co-occurrence of physical frailty and cognitive impairment in Chinese community-dwelling older adults | Xie, B;Ma, C;Chen, Y  Wang, J | 10.1111/hsc.13092 | Without interest outcomes |
| 23 | Kunicki, Z. J. 2023 | Prevalence of Comorbid Depression and Insomnia Among Veterans Hospitalized for Heart Failure with Alzheimer Disease and Related Disorders. | Kunicki, Z J;Frietchen, R  McGeary, J E;Jiang, L  Duprey, M S;Bayer, T  Singh, M;Primack, J M  Kelso, C M;Wu, W C  Rudolph, J L;Bozzay, M L | 10.1016/j.jagp.2023.01.026 | Without interest outcomes |
| 24 | Kronholm, E.2009 | Self-reported sleep duration and cognitive functioning in the general population | Kronholm, E;Sallinen, M  Suutama, T;Sulkava, R  Era, P;Partonen, T | 10.1111/j.1365-2869.2009.00765.x | Without interest outcomes |
| 25 | Jaussent, I. 2012 | Sleep complaints and cognitive decline in communitydwelling elderly: An 8-year prospective study. | Jaussent, I;Bouyer, J  Ancelin, M;Berr, C  Foubert-Samier, A  Ritchie, K;Ohayon, M  Besset, A;Dauvilliers, Y | 10.1111/j.1365-2869.2012.01044.x | Without interest outcomes |
| 26 | Lee, S.2023 | Sleep Disorders and Cognitive Aging among Cognitively Impaired vs. Unimpaired Older Adults | Lee, S;Nelson, M E  Hamada, F;Wallace, M L  Andel, R;Buxton, O M  Almeida, D M;Lyketsos, C  Small, B J | 10.1093/geront/gnad152 | Without interest outcomes |
| 27 | Sindi, S. 2018 | Sleep disturbances and later cognitive status: a multi-centre study. | Sindi, S;Johansson, L  Skoog, J;Mattsson, A D  Sjöberg, L;Wang, H X  Fratiglioni, L;Kulmala, J  Soininen, H;Solomon, A  Johansson, B;Skoog, I  Kivipelto, M;Kåreholt, I | 10.1016/j.sleep.2017.11.1149 | Without interest outcomes |
| 28 | Chen, J C.2016 | Sleep duration, cognitive decline, and dementia risk in older women | Chen, J C;Espeland, M A  Brunner, R L;Lovato, L C  Wallace, R B;Leng, X  Phillips, L S;Robinson, J G;Kotchen, J M  Johnson, K C;Manson, J E  Stefanick, M L;Sarto, G E  Mysiw, W J | 10.1016/j.jalz.2015.03.004 | Without interest outcomes |
| 29 | Eun, H. 2011 | The risk of dementia by sleep disturbances in communitydwelling elderly population of urban and island areas | Eun, H  Kim, T | 10.1016/S1876-2018(11)60207-4 | Without interest outcomes |
| 30 | Cricco, Meredith.2001 | The impact of insomnia on cognitive functioning in older adults | Cricco, Meredith  Simonsick, Eleanor M  Foley, Daniel J |  | Without interest outcomes |
| 31 | Cavaillès, C.2020 | Complaints of poor sleep, daytime sleepiness, hypnotic use and risk of dementia: A longitudinal study in the general elderly population | Cavaillès, C; Berr, C; Helmer, C; Gabelle, A  Jaussent, I; Dauvilliers, Y | 10.1002/alz.12278 | Conference Abstract |
| 32 | Mason, B. 2022 | Decrease risk of 2-year incidence of Alzheimer’s disease among older adults who report sleep symptoms | Mason, B; Wills, C  Tubbs, A; Seixas, A  Turner, A; Jean-Louis, G  Killgore, W; Grandner, M | 10.1093/sleep/zsac079.270 | Conference Abstract |
| 33 | Baek, M S.2019 | Insomnia increase the risk of alzheimer’s disease and vascular dementia: a nationwide population-based study in south korea. | Baek, M S; Han, K  Koo, Y; Choi, B K  Na, H K; Lyoo, C H  Cho, H | 10.1016/j.jalz.2019.06.2694 | Conference Abstract |
| 34 | Tsai, M K.2017 | Self-reported sleep disturbances and cognitive decline in the elderly: Attenuating the risk by physical activity | Tsai, M K;Chen, J H  Chiou, J M;Chen, T F  Chen, Y F;Tang, S C  Yeh, S J;Chiu, M J  Chen, Y C |  | Conference Abstract |
| 35 | Berr, C. 2012 | Sleep and cognitive decline in the elderly: The French three-city cohort | Berr, C; Jaussent, I  Bouyer, J; Ancelin, M L  Foubert, A S; Ritchie, K  Ohayon, M M; Besset, A  Dauvilliers, Y | 10.1016/j.jalz.2012.05.618 | Conference Abstract |
| 36 | Bubu, O M. 2016 | Sleep disorders and Alzheimer's dementia: A cross-sectional analysis of older adults discharged from Tampa General Hospital | Bubu, O M;Mortimer, J  Schwartz, S;Wu, Y  Anderson, W;Morgan, D |  | Conference Abstract |
| 37 | Kuhler, C.2022 | Sleep disorders as a potential risk factor for dementia in elderly adults | Kuhler, C;Wills, C  Mason, B;Tubbs, A  Seixas, A;Turner, A  Jean-Louis, G  Killgore, W;Grandner, M | 10.1093/sleep/zsac079.271 | Conference Abstract |
| 38 | Yaffe, K.2014 | Sleep disturbance and risk of dementia among older veterans | Yaffe, K;Nettiksimmons, J  Byers, A L |  | Conference Abstract |
| 39 | Huang, T H.2014 | Status of associated factors for the quality of sleep in patients with dementia: An epidemiological assessment | Huang, T H;Chang, C E  Yang, T; Chou, Y C  Lin, S C; Sun, C A | 10.1017/S1461145714000741 | Conference Abstract |
| 40 | Osorio, R S.2011 | Greater risk of Alzheimer's disease in older adults with insomnia. | Osorio, R S;Pirraglia, E  Agüera-Ortiz, L F  During, E H;Sacks, H  Ayappa, I;Walsleben, J  Mooney, A;Hussain, A  Glodzik, L;Frangione, B  Martínez-Martín, P  De Leon, M J | 10.1111/j.1532-5415.2010.03288.x | Conference Abstract |
| 41 | Merlino, G.2010 | Daytime sleepiness is associated with dementia and cognitive decline in older Italian adults: a population-based study | Merlino, G; Piani, A  Gigli, G L; Cancelli, I  Rinaldi, A; Baroselli, A  Serafini, A; Zanchettin, B  Valente, M | 10.1016/j.sleep.2009.07.018 | Conference Abstract |
| 42 | Spira, A P. 2013 | Self-reported sleep and β-amyloid deposition in community-dwelling older adults | Spira, A P; Gamaldo, A A  An, Y; Wu, M N  Simonsick, E M;Bilgel, M  Zhou, Y; Wong, D F  Ferrucci, L; Resnick, S M | 10.1001/jamaneurol.2013.4258 | Conference Abstract |
