# Supplementary material for: Insomnia and risk of all-cause dementia: A systematic review and meta-analysis
Source: PLoS One. 2025 Apr 9;20(4):e0318814. doi: 10.1371/journal.pone.0318814 (PMC11981150; doi:10.1371/journal.pone.0318814)
Supplement: S6 Table — (DOC) [file pone.0318814.s014.doc]

**Supplementary Table S6. The quality assessment of cohort and case-control studies.**

| Study | Year | Selection | Comparability | Outcome | Total |
| --- | --- | --- | --- | --- | --- |
| Cohort studies (n=11) | | | | | |
| Selbæk-Tungevåg, S | 2023 | *** | ** | *** | 8 |
| Tan, X | 2023 | *** | ** | *** | 8 |
| Wong, R | 2023 | *** | ** | *** | 8 |
| Cavaillès, C | 2022 | *** | ** | *** | 8 |
| Robbins, R | 2021 | *** | ** | ** | 7 |
| Resciniti, N.V | 2021 | *** | ** | ** | 7 |
| Sindi, S | 2018 | *** | ** | ** | 7 |
| Yaffe, K | 2015 | **** | ** | *** | 9 |
| Chen, P.L | 2012 | *** | ** | ** | 7 |
| Elwood, P.C | 2010 | *** | ** | ** | 7 |
| Foley, D | 2001 | *** | ** | ** | 7 |
| Case-control studies (n=5) | | | | | |
| Lin, W | 2023 | **** | ** | ** | 8 |
| Baek, M.S | 2021 | **** | ** | * | 7 |
| Hoile, R | 2019 | **** | ** | ** | 8 |
| Hung, C.M | 2018 | **** | ** | ** | 8 |
| Morgan, K | 1994 | **** | * | *** | 8 |

The NOS scale was used to evaluate the quality of the cohort and case-control studies.
