# Supplementary material for: Insomnia and risk of all-cause dementia: A systematic review and meta-analysis
Source: PLoS One. 2025 Apr 9;20(4):e0318814. doi: 10.1371/journal.pone.0318814 (PMC11981150; doi:10.1371/journal.pone.0318814)
Supplement: Attachment — Submitted filename: Response to Reviewers.doc [file pone.0318814.s017.doc]

**Response to reviewers**

**Dear reviewers and editors:**

On behalf of my co-authors, we thank you very much for your letter and comments on our manuscript entitled "***Insomnia and Risk of All-Cause Dementia: A Systematic Review and Meta-Analysis***" (Manuscript ID: PONE-D-24-20907). We appreciate the editors and reviewers for their constructive and valuable comments. We have revised our manuscript considerably according to the editors’ and reviewers’ comments, questions, and suggestions. In the event that we missed any one of the comments please let us know. This document includes our responses to reviewers and editor comments point by point, and the revised portion are marked in RED in our manuscript.

# Reply to reviewer 1

While we understand that the manuscript we submitted did not fully align with your standards and requirements, we have carefully addressed these concerns and issues you raised in our revised version. We would be grateful if you could consider our manuscript once more.

**Comment 1:** The article fails on many levels to be considered for publication.

**Reply 1:** We sincerely appreciate your critical and constructive feedback. We acknowledge that the manuscript has limitations and areas for improvement. However, under your guidance, as well as that of another reviewer, we have undertaken a thorough review and revision. As a result, we believe the manuscript has been significantly improved, particularly in the areas of bias risk and heterogeneity.

**Comment 2:** For instance, the risk and domains of bias are not elaborately explained.

**Reply 2:** We greatly appreciate your comments regarding the assessment of bias risk and bias domains in our manuscript, and we acknowledge that this section may not have been sufficiently detailed in the previous submission.

We used the Newcastle-Ottawa Scale (NOS) to systematically assess the risk of bias in the included observational studies. The NOS scale is a widely used tool for systematic reviews and meta-analyses, designed specifically to evaluate the quality of non-randomized studies (such as cohort and case-control studies) (obtain from:https://www.ohri.ca/programs/clinical_epidemiology/oxford.asp)[1]. It assesses studies across three main domains: **selection bias, comparability bias, and outcome assessment bias**. Each domain contains several specific items, and the overall score ranges from 0 to 9 points, with higher scores indicating a lower risk of bias. In order to clearly understand the content of the NOS scale assessment, we uploaded the original NOS scale in the S**upplementary Table S1** of the revised manuscript.

Using the NOS, we systematically assessed the risk of bias in the included studies, ensuring a thorough and objective evaluation of their quality of methodology. The specific scores of each study in the three domains are detailed in **Supplementary Table S4**. Additionally, to enhance the transparency of the manuscript, we have included a **Bias Domains Heatmap for Included Studies (Figure 2 of revised manuscript)**, which visually presents each study's scores in the respective bias domains.

We hope that the revised bias risk assessment and the additional explanations in the manuscript will address your concerns and meet the required standards. Thank you for your consideration, and we look forward to your feedback about this part.

**Changes in the text:**

**Page 9, Line 246~253**:

“Independently, MMX and SXM applied the NOS to assess each included study, emphasizing three critical quality dimensions related to selection bias, Comparability Bias, and Outcome Assessment Bias. Studies scoring 7-9 points on the Newcastle-Ottawa Scale were considered high quality with low risk of bias, 4-6 points indicated medium quality with moderate risk of bias, and 0-3 points represented lower quality with high risk of bias. **Supplementary Table S4** exhibited the specific items of the NOS quality assessment form for non-randomized controlled trials.’’

**Page 12, Line 310~314**: **Figure 2** gives a heatmap that illustrates the distribution of bias across the included studies, highlighting the frequency of high, moderate, and low risk of bias in various domains assessed using the Newcastle-Ottawa Scale. Each cell represents the proportion of studies falling into each risk category for a specific domain, with color gradients used to indicate varying levels of bias.

**Page 31, Line 702~705**: **Figure 2 Bias Domains Heatmap for Included Studies.** The heatmap is color-coded, with darker shades indicating higher risks of bias and lighter shades representing lower risks. The domains evaluated include selection, comparability, and outcome/exposure assessment, with high scores categorized as lower risk.

**Comment 3:** The discussion section also appears to be compromised.

**Reply 3:** Thank you for your valuable feedback regarding the discussion section. We understand your concern that the discussion may not have fully addressed key aspects of the study. In response, we have carefully revisited this section and made the following improvements:

Clarified the main findings: We have provided a clearer summary of the key results and how they align with or differ from previous research in the field.

Addressed the implications: We have expanded the discussion on the clinical and scientific implications of our findings, explaining how they contribute to the current understanding of dementia and how they might inform future research or clinical practice.

Considered the limitations: We have explicitly acknowledged the limitations of the study, including potential biases, confounding factors, and generalizability issues, and discussed how they may affect the interpretation of the results.

Provided suggestions for future research: We have added recommendations for future studies that could address the limitations of our work and explore new avenues based on our findings.

We hope that these revisions strengthen the discussion section and provide a more thorough and balanced interpretation of the results. We would greatly appreciate your further feedback on these changes.

We truly appreciate the time and effort you have taken to carefully review our manuscript. After revising and restructuring the discussion section based on your feedback, we are hopeful for another thorough evaluation from you. We kindly request more specific suggestions, as general feedback makes it difficult for us to make targeted improvements that would meaningfully enhance the quality of our manuscript.

**Changes in the text:**

**Page 18, Line 405~406**:

“However, the subtypes of insomnia, sleep initiation difficulties, sleep maintenance disorders, and early morning awakening were not associated with dementia.”

**Page 19-21, Line 425~462**:

“Drawing upon the findings of this meta-analysis and integrating them with existing evidence, we posit that insomnia is linked to a heightened risk of all-cause dementia, AD and VD. However, the precise mechanisms underlying this association remain elusive.

**Amyloid-Beta Clearance and Insomnia**

One of the key mechanisms linking insomnia to an increased risk of Alzheimer’s disease (AD) is the impaired clearance of amyloid-beta (Aβ), a hallmark of AD[42]. During sleep, the brain’s glymphatic system is more active, facilitating the removal of metabolic waste products like Aβ[43-45]. Studies have shown that Aβ clearance is significantly reduced during wakefulness, leading to its accumulation over time [20]. Chronic sleep deprivation, which often accompanies insomnia, may exacerbate this process, resulting in increased Aβ deposition in the brain[46]. This accumulation is believed to accelerate the onset and progression of AD, suggesting that sleep plays a critical role in neuroprotection.

**Tau Protein Abnormalities and Sleep Disruption**

In addition to Aβ, abnormalities in Tau protein are another significant factor in AD pathogenesis [47]. Disrupted sleep has been shown to elevate Tau levels in both animal models and human cerebrospinal fluid (CSF), which may drive neurodegenerative changes. The study by Holth and his colleagues [21] confirmed that the sleep-wake cycle regulates the level of Tau protein in interstitial fluid, which increased by about 90% during wakefulness and by about 100% during sleep deprivation in mice, and by more than 50% during sleep deprivation in human cerebrospinal fluid (CSF), compared with the level of the protein during sleep. Given that Tau protein aggregates are a key feature of AD, chronic sleep disturbances may amplify Tau pathology, accelerating cognitive decline. The study by Rothman [48] further validated this in an Alzheimer's disease mouse model, where sleep restriction (6 hours per day for 6 weeks) exacerbated memory deficits and increased Aβ and Tau levels in the cortex compared to controls. This provides further evidence that sleep deprivation can significantly contribute to both Aβ and Tau accumulation, highlighting the dual impact of sleep on AD pathology.

**Inflammation, Vascular Dysfunction, and Insomnia**

The association between insomnia and vascular dementia (VD) appears to be even stronger than that with AD, suggesting that vascular mechanisms may also be crucial. Chronic sleep loss is known to activate inflammatory pathways, such as nuclear factor kappa-B (NF-κB) and activator of transcription (STAT) family proteins, which can lead to vascular damage and increased risk of cerebrovascular disease [49, 50]. Additionally, insomnia has been associated with white matter hyperintensities, which are markers of cerebrovascular dysfunction and are commonly observed in patients with VD [51]. This vascular pathway may explain the particularly strong link between insomnia and VD observed in our analysis, indicating that sleep disturbances could exacerbate both neurodegenerative and vascular pathways contributing to dementia.”

**Page 23-24, Line 500~513：**

“The subgroup analysis based on sample size revealed contrasting results. Studies with a sample size of Greater than or equal to 10,000 indicated a significant association between insomnia and all-cause dementia, whereas studies with fewer than 10,000 participants showed no such association, suggesting potential variability in the observed effects depending on study size. Finally, we did subgroup analyses based on insomnia diagnostic criteria demonstrated that the risk of dementia associated with insomnia was higher in studies using ICD codes for diagnosis compared to those using self-reported diagnoses. This suggests that the method of diagnosis may influence the strength of the observed association between insomnia and risk of dementia. Although most studies diagnose insomnia using questionnaires, such as the National Health and Nutrition Examination Survey (NHANES) in the United States and the UK Biobank, questionnaires may introduce bias due to participant forgetfulness or deliberate concealment. This could potentially weaken the observed association between insomnia and the risk of all-cause dementia.”

**Comment 4:** The heterogeneity in the means of diagnosis of insomnia is another source of concern;

**Reply 4:** Thank you for raising this important point regarding the heterogeneity in the means of diagnosing insomnia. We agree that variability in diagnostic criteria can contribute to heterogeneity and potentially impact the results of the meta-analysis.

Regarding the sources of heterogeneity, we conducted a comprehensive assessment. Specifically, we explored factors such as the geographical location of the included studies, sample size, follow-up duration, the diagnostic criteria for both exposure and outcomes, and study design. Notably, we classified the studies according to insomnia diagnostic standards, including ICD codes, self-reported insomnia, and DSM criteria.

Following these steps, we further investigated the sources of heterogeneity using standard approaches [2]. First, we applied a random-effects model; second, we performed sensitivity analyses and subgroup analysis; and finally, we conducted a multivariable meta-regression analyses to explore potential sources of heterogeneity. All results indicated that our findings are robust.

A Multivariate meta-regression analysis was also performed to explore the potential sources of heterogeneity in the relationship between insomnia and dementia risk. The model included the following covariates: continent, follow-up duration, number of participants, study type, and insomnia diagnosis criteria and dementia diagnosis criteria. The results showed that all covariates, including continent (p = 0.924), follow-up years (p = 0.524), number of participants (p = 0.984), study type (p = 0.527), and insomnia diagnosis criteria (p = 0.621), and dementia diagnosis criteria (p = 0.623) did not reach statistical significance. However, the overall association between insomnia and All-Cause dementia remains robust.

We have included a detailed discussion of these findings in limitations and prospection section of the revised manuscript, along with a more cautious interpretation of the results given the persistent residual heterogeneity. We hope this additional analysis addresses your concern, and we look forward to your further feedback.

**Comment 5:** Was heterogeneity within limits to allow for a meta-analysis?

**Reply 5:** Thank you for your valuable comments on the topic of Heterogeneity within the range that allowed for meta-analysis.

Through an extensive review of the literature, we identified that heterogeneity in meta-analyses generally arises from three sources: **clinical heterogeneity, methodological heterogeneity, and statistical heterogeneity [2].**

a. **Clinical heterogeneity** refers to differences in participant demographics, measurement of exposures and outcomes, or other clinical factors across studies[3].
b. **Methodological heterogeneity** refers to differences in study design, data collection, and analysis methods, such as study type, follow-up duration, and statistical techniques[3].
c. **Statistical heterogeneity** arises from quantitative differences in reported effect sizes (e.g., OR, RR, mean difference). Even when studies examine similar populations and interventions, effect sizes may vary considerably, as reflected by high I² values. For example, in studies examining the association between insomnia and dementia risk, the odds ratios (ORs) ranged from 1.2 to 3.5, representing statistical heterogeneity.

In our study, we assessed statistical heterogeneity using I² statistics and the Q-test. The high *I*²values observed in the meta-analysis indicate significant variability in effect sizes between studies, which reflects statistical heterogeneity.

Statistical heterogeneity is a result of clinical or methodological heterogeneity or the combination of two heterogeneities among the studies [2]. It is important to emphasize that clinical and methodological heterogeneity are **inevitable in any meta-analysis result [4]**. The studies we included differed in terms of participant characteristics, measurement of exposures and outcomes, study design, data collection, and analytical methods. If all studies were completely clinically and methodologically identical, the significance of a meta-analysis would be greatly diminished.

In another part, in the presence of heterogeneity, creative exploration of sources of statistical heterogeneity may allow us to identify subpopulations of patients who are particularly vulnerable to adverse events [5]. In this context, the presence of heterogeneity may be advantageous when applying the evidence to individual patients [2]. For example, when we combined insomnia and all-cause dementia risk, the heterogeneity was large. However, we found that when we divided the subgroups according to the geographical location (continent) of the study, the heterogeneity was greatly reduced, which may have specifically discovered the geographical differences in insomnia for all-cause dementia or the cultural and racial differences behind it.

To recognize these potential sources of heterogeneity, we performed subgroup analyses and meta-regression analyses. We elaborate on the sources of heterogeneity and their significance in the subgroup analysis section and meta-regression section of the revised manuscript. Suggestions for future research are outlined in the limitations and outlook section. Specifically, we recommend standardizing diagnostic criteria in future studies to reduce heterogeneity and its potential impact on meta-analysis results.

Therefore, considering all these factors and combined with the results of sensitivity analyses, we believe that our meta-analysis results are robust. Although we advocate for cautious interpretation of our findings, they still provide conservative estimates of the association between insomnia and all-cause dementia risk. Our analysis does not claim to be immediately applicable to the clinic, but rather provides reliable reference values for future research in this field based on reasonable and robust analysis.

**REFERENCE**

[1] Wells GA, Shea B, O’Connell D, Peterson J, Welch V, Losos M, et al. The Newcastle-Ottawa Scale (NOS) for assessing the quality of nonrandomised studies in meta-analyses. 2000.

[2] Schulzke S. Assessing and exploring heterogeneity[M]//Principles and practice of systematic reviews and meta-analysis. Cham: Springer International Publishing, 2021: 33-41.

[3] Cochrane. Exploring heterogeneity—slidecast. http://training.cochrane.org/resource/exploringheterogeneity. Accessed 27 June 2017

[4] Higgins JP. Commentary: Heterogeneity in meta-analysis should be expected and appropriately quantified. Int J Epidemiol. 2008;37(5):1158–60.

**[5]** Fletcher J. What is heterogeneity and is it important? BMJ. 2007;334(7584):94–6.

**Changes in the text:**

**Page 10, Line 266~269**:

“Given the complex nature of insomnia and all-cause dementia, subgroup analyses based on continent, follow-up duration, gender, number of participants, study type, insomnia diagnostic criteria, and dementia diagnostic criteria will be performed. ”

**Page 10, Line 271~276**:

“Random-effects multivariable meta-regression analyses were performed to investigate potential sources of heterogeneity and to assess the impact of moderators, including Continent, follow-up duration, number of participants, study type, insomnia diagnostic criteria and dementia diagnostic criteria.”

**Page 14, Line 340~358:**

**“Subtypes of Insomnia and Risk of All-Cause Dementia**

Underlying insomnia subtypes, five studies [16, 23, 25, 26, 39] examined the effectiveness of the link between initial insomnia and risk of all-cause dementia. Pooled analysis findings point to no statistically significant association Between Initial Insomnia and risk of all-cause dementia (OR=1.01; 95%CI=0.77-1.31; I2=83.4%; P=0.001). The results of forest plot were shown in **Supplement Figure S6**. The sensitivity analysis demonstrates the robustness of the meta-analysis results, as presented in **Supplementary Figure S7**.

Apart from this, four studies [16, 23, 25, 26] research on the relationship between sleep-maintenance insomnia and risk of all-cause dementia. After pool analysis, there is no evidence to suggest that sleep-maintenance insomnia is linked to the risk of developing all-cause dementia (OR=0.88; 95%CI=0.66-1.17; I2=90.1%; P=0.001). The forest plot results are displayed in **Supplementary Figure S8**. The robustness of the meta-analysis results is confirmed by the sensitivity analysis, provided in **Supplementary Figure S9**.

Additionally, pool analysis under two studies [16, 25] also showed that early morning awakening was not associated with the risk of all-cause dementia (OR=0.94; 95%CI=0.83-1.07; I2=0; P=0.939). The forest plot results were depicted in **Supplementary Figure S10**, providing an overview of the individual study estimates and pooled effect size. **Supplementary Figure S11** presents the sensitivity analysis, which verified the stability and reliability of the meta-analysis findings.”

**Page 15, Line 359~368**:

“Meta regression

A Multivariate meta-regression analysis was performed to explore the potential sources of heterogeneity in the relationship between insomnia and dementia risk. The model included the following covariates: continent, follow-up duration, number of participants, study type, insomnia diagnosis criteria, and dementia diagnosis criteria.

The results showed that no covariates was significantly associated with the effect size, including continent (p = 0.924), follow-up years (p = 0.524), number of participants (p = 0.984), study type (p = 0.527), and insomnia diagnosis criteria(p = 0.621), and dementia diagnosis criteria (p = 0.623), did not reach statistical significance. The details of meta-regression results with the risk of All-cause dementia are depicted in **Table 3**. ”

**Page 16-17, Line 380~394**: In the results section. Added:

“Subgroup analysis based on study type indicated that retrospective studies[16, 27, 28, 38-40] (OR=1.45) and prospective studies[17, 22] (OR=1.17). According to the number of participants, the subgroup analysis was performed, with the number of participants greater than or equal to 10,000[22, 27, 28, 38, 40] (OR=1.53) and the number of participants less than 10,000[16, 17, 39] (OR=1.17). For the subgroup analysis based on insomnia diagnostic criteria, patients were classified into three subgroups: DSM criteria [16], self-report [17, 22, 27, 39], and ICD codes [28, 38, 40]. The results for the DSM criteria subgroup did not show a significant association with all-cause dementia. ICD codes diagnoses showed a stronger association between insomnia the risk of All-Cause dementia risk (OR=1.89) than self-report (OR=1.11). Furthermore, subgroup analysis of dementia diagnosis indicated DSM criteria subgroup [16, 17, 39] was no significant association with all-cause dementia. ICD codes diagnoses [22, 28, 38, 40] showed a stronger association between insomnia the risk of All-Cause dementia risk (OR=1.68) than self-report [27] (OR=1.05). The results of the subgroup analysis for the risk of all-cause dementia in patients with insomnia are summarized in **Table 2**.”

**Page 18, Line 405~406**:

“However, the subtypes of insomnia, sleep initiation difficulties, sleep maintenance disorders, and early morning awakening were not associated with dementia.”

**Page 23-24, Line 500~513：**

“The subgroup analysis based on sample size revealed contrasting results. Studies with a sample size of Greater than or equal to 10,000 indicated a significant association between insomnia and all-cause dementia, whereas studies with fewer than 10,000 participants showed no such association, suggesting potential variability in the observed effects depending on study size. Finally, we did subgroup analyses based on insomnia diagnostic criteria demonstrated that the risk of dementia associated with insomnia was higher in studies using ICD codes for diagnosis compared to those using self-reported diagnoses. This suggests that the method of diagnosis may influence the strength of the observed association between insomnia and risk of dementia. Although most studies diagnose insomnia using questionnaires, such as the National Health and Nutrition Examination Survey (NHANES) in the United States and the UK Biobank, questionnaires may introduce bias due to participant forgetfulness or deliberate concealment. This could potentially weaken the observed association between insomnia and the risk of all-cause dementia.”

**Page 24, Line 516~519**:

“We used a random-effects model to combine all effect sizes. Sensitivity analyses were performed to verify the robustness of the findings. We also used subgroup analyses and meta-regression to explore the sources of heterogeneity.”

**Page 24, Line 521~528：**

“Clinical heterogeneity and methodological heterogeneity due to differences in demographics and experimental designs are the main sources of statistical heterogeneity in this meta-analysis and cannot be completely avoided. Although we did not identify possible sources of heterogeneity, we speculate that a key limitation is the reliance on self-reported insomnia, which could lead to recall bias or misclassification. Additionally, the considerable heterogeneity observed suggests that other unmeasured factors could influence the outcomes. Therefore, the results of our meta-analysis should be interpreted with caution.”

**Page 24-25, Line 528~533**:

“Future research should prioritize the use of standardized diagnostic criteria for insomnia and incorporate objective sleep measures to reduce heterogeneity. Long-term follow-up studies, particularly those exceeding five years, are necessary to fully capture the chronic impact of insomnia on dementia risk. Additionally, large-scale, multi-center studies adjusting for key confounders will help identify at-risk populations and clarify the role of insomnia as a modifiable risk factor for dementia.”

**Comment 6:** Were all studies included for systematic review as well as for meta-analysis?

**Reply 6:** Thank you for your thoughtful question regarding the inclusion of studies in our meta-analysis.

In the studies we included, multiple exposures and corresponding outcome measures were reported across different clinical studies. Not every study addressed all outcome measures, but several studies included multiple outcomes, which allowed us to use them in more than one analysis.

After a detailed literature screening process, we included 16 original studies. However, for different meta-analyses addressing specific exposures and outcomes, we included different studies. This approach was taken to ensure the homogeneity of studies in each meta-analysis, as part of our strategy to minimize heterogeneity.

To avoid any potential confusion, we have revised the Results section of the manuscript for clarity. We hope this adjustment helps to better convey the structure of our meta-analyses.

Firstly, in the meta-analyses addressing the association between insomnia and the risk of all-cause dementia, we conducted separate analyses for different outcomes:

a. Insomnia and risk of all-cause dementia
b. Insomnia and risk of Alzheimer's disease (AD)
c. Insomnia and risk of vascular dementia (VD)

Additionally, for the meta-analyses on insomnia subtypes and the risk of all-cause dementia, we performed separate analyses for different insomnia subtypes:

a. Difficulty initiating sleep and risk of all-cause dementia
b. Difficulty maintaining sleep and risk of all-cause dementia
c. Early morning awakening and risk of all-cause dementia

By stratifying the analyses based on different outcomes and insomnia subtypes, we aimed to improve the clarity of the results and ensure that each meta-analysis was based on a more homogeneous set of studies. We hope this explanation clarifies our approach and that the revisions in the manuscript resolve any concerns.

**Changes in the text:**

**Page 13, Line 315**: added: “**Insomnia and Risk of All-Cause Dementia**”

**Page 14-15, Line 340~358**: added:

“**Subtypes of Insomnia and Risk of All-Cause Dementia**

Underlying insomnia subtypes, five studies [16, 23, 25, 26, 39] examined the effectiveness of the link between initial insomnia and risk of all-cause dementia. Pooled analysis findings point to no statistically significant association Between Initial Insomnia and risk of all-cause dementia (OR=1.01; 95%CI=0.77-1.31; I2=83.4%; *P*=0.001). The results of forest plot were shown in **Supplement Figure S5**. The sensitivity analysis demonstrates the robustness of the meta-analysis results, as presented in **Supplementary Figure S6**.

Apart from this, four studies [16, 23, 25, 26] research on the relationship between sleep-maintenance insomnia and risk of all-cause dementia. After pool analysis, there is no evidence to suggest that sleep-maintenance insomnia is linked to the risk of developing all-cause dementia (OR=0.88; 95%CI=0.66-1.17; I2=90.1%; P=0.001). The forest plot results are displayed in **Supplementary Figure S7**. The robustness of the meta-analysis results is confirmed by the sensitivity analysis, provided in **Supplementary Figure S8**.

Additionally, pool analysis under two studies [16, 25] also showed that early morning awakening was not associated with the risk of all-cause dementia (OR=0.94; 95%CI=0.83-1.07; I2=0; P=0.939). The forest plot results were depicted in **Supplementary Figure S9**, providing an overview of the individual study estimates and pooled effect size. **Supplementary Figure S10** presents the sensitivity analysis, which verified the stability and reliability of the meta-analysis findings.”

**Comment 7:** The figures are often given as a passing reference and not adequately explained.

**Reply 7:** Thank you for your insightful feedback regarding the figures in the manuscript. We recognize that some of the figures may not have been adequately explained in the original submission.

In response to your comment, we have thoroughly revised the Results section to provide more detailed explanations of the figures. Specifically, we have:

Enhanced the descriptions of each figure to clearly highlight the key findings and their relevance to the overall analysis.

Integrated the figures more closely with the text, ensuring that each figure is directly referenced and its results are fully explained in the context of the study.

Clarified the statistical details and visual representation in each figure, so that readers can better understand how the data supports our conclusions.

We hope that these revisions address your concerns and provide clearer insights into the data presented in the figures. We look forward to your further feedback

**Changes in the text:**

**Page 13, Line 321~324**:

“**Figure 3** presents the forest plot illustrating the association between insomnia and the risk of all-cause dementia, demonstrating a pooled odds ratio of 1.36 (95% CI: 1.01-1.84) with significant heterogeneity (*I*²=98.7%).”

**Page 13, Line 331~332**:

**“Figure 4** demonstrates the forest plot of insomnia and risk of AD, given a pooled odds ratio of 1.52 (95% CI: 1.19-1.93) with significant heterogeneity (*I*²=65.8%).”

**Page 14, Line 337~339**:

“The forest plot results are depicted in **Supplement** **Figure S4,** illustrated the pooled odds ratio of 1.52 (95% CI: 1.19-1.93) with significant heterogeneity (I²=65.8%).”

**Page 31-32, Line 694~726**: Figure Legends section added:

“**Figure legends**

**Figure 1** **PRISMA flow diagram illustrating the study selection process for the meta-analysis on the association between insomnia and the risk of All-Cause dementia.** The flow diagram outlines the number of records identified through database searches (PubMed, Embase, Cochrane Library), the number of records screened after removing duplicates, and the number of full-text articles assessed for eligibility. It also shows the number of studies excluded at each stage, with reasons for exclusion provided during full-text assessment. Finally, the diagram highlights the total number of studies included in the qualitative synthesis and those included in the quantitative synthesis (meta-analysis).

**Figure 2** **Bias Domains Heatmap for Included Studies.** The heatmap is color-coded, with darker shades indicating higher risks of bias and lighter shades representing lower risks. The domains evaluated include selection, comparability, and outcome/exposure assessment, with high scores categorized as lower risk.

**Figure 3 Sensitive analysis plot of the relationship between Insomnia and risk of Dementia.** Sensitivity analysis plot showing the impact of each individual study on the overall pooled effect size in the meta-analysis examining the relationship between insomnia and the risk of dementia. Each point represents the recalculated pooled odds ratio (OR) after omitting one study at a time. The horizontal line represents the 95% confidence interval (CI) of the overall pooled OR. The plot demonstrates that the exclusion of any single study does not significantly alter the overall effect size, indicating the robustness of the meta-analysis results.

**Figure 4** **Forest plot illustrating the association between insomnia and the risk of all-cause dementia.** The plot displays the individual effect sizes (Odds Ratios, ORs) and 95% confidence intervals (CIs) for each study included in the meta-analysis. The solid squares represent the ORs for each study, with the size of the square proportional to the weight of the study in the analysis. The horizontal lines correspond to the 95% CIs. The diamond at the bottom represents the overall pooled OR and its 95% CI, calculated using a random-effects model. An OR greater than 1 indicates an increased risk of dementia associated with insomnia.

**Figure 5** **Funnel plot assessing potential publication bias in studies examining the association between insomnia and the risk of dementia.** Each point represents an individual study included in the meta-analysis. The x-axis shows the effect size (Odds Ratios, ORs), and the y-axis represents the standard error of the log OR, a measure of study precision. Studies with larger sample sizes and greater precision appear toward the top of the plot, while smaller, less precise studies are near the bottom. Symmetry of the plot indicates the absence of publication bias, whereas asymmetry suggests potential bias or heterogeneity. The vertical dashed line represents the pooled effect size from the meta-analysis.”

Under your kind help and professional guidance, we believe that our manuscript has been improved substantially. As researchers, we all strive to impress the readers more comprehensive and more important information. We are looking forward to your further suggestions.

# Reply to reviewer 2

**Comment 1:** The manuscript titled "Insomnia and Risk of All-Cause Dementia: A Systematic Review and Meta-Analysis" addresses a pertinent issue in the field of sleep disorders and neurodegenerative diseases. The study aims to elucidate the association between insomnia and the risk of developing dementia, including Alzheimer's disease (AD) and Vascular dementia (VD). The manuscript is well-structured, and the research question is clearly articulated. The authors have conducted an extensive and systematic literature search and have utilized appropriate statistical methods to synthesize the findings from multiple studies.

**Reply 1:** Thank you for your positive feedback on our manuscript titled "Insomnia and Risk of All-Cause Dementia: A Systematic Review and Meta-Analysis." We greatly appreciate your recognition of the importance of this topic, as well as your acknowledgment of the clear structure, comprehensive literature search, and appropriate statistical methods used in our study.

We are pleased that you found the research question clearly articulated and the methods sound, as it was our goal to provide a thorough and rigorous investigation of the association between insomnia and dementia, including Alzheimer's disease (AD) and vascular dementia (VD). Your comments further motivate us to continue refining our work to ensure its clarity and impact on the field.

If you have any further questions about the manuscript, please do not hesitate to contact us. We welcome further discussions. We look forward to your additional feedback.

**Comment 2:** Technical Soundness and Data Support: The manuscript is technically sound in many respects, and the data presented do support the authors' conclusions to a certain extent. The meta-analysis incorporates 16 studies, involving over 9 million individuals, which is commendable. The inclusion criteria are well-defined, and the studies included in the analysis are generally of high quality, as indicated by the Newcastle-Ottawa Quality Rating Scale (NOS) scores.

**Reply 2:** Thank you for your thoughtful feedback and for recognizing the technical soundness of our manuscript. We are pleased that you found the data and conclusions to be well-supported.

We also appreciate your positive comments on the clear inclusion criteria and the overall high quality of the studies included in the analysis, as reflected in their Newcastle-Ottawa Quality Rating Scale (NOS) scores. Ensuring the technical rigor and accuracy of the data was a key priority for us, and we are glad that these aspects of the manuscript have been well received.

Should you have any further suggestions or areas you believe require additional clarification, we would be happy to address them. We look forward to your continued feedback.

**Comment 3:** Heterogeneity: One of the major concerns is the significant heterogeneity observed in the analysis, particularly in studies with shorter follow-up periods (less than five years). The high *I*² values in several analyses suggest that there is substantial variability among the studies, which may not be fully accounted for by the random-effects model used. The authors have performed sensitivity analyses to assess the robustness of their findings, but the persistence of heterogeneity indicates that the results should be interpreted with caution. It would be beneficial to explore the potential sources of this heterogeneity further, such as differences in population characteristics, study designs, or diagnostic criteria for insomnia and dementia.

**Reply 3:** Thank you for your insightful comments regarding the heterogeneity observed in our analysis. We fully acknowledge the significance of this issue, and appreciate the opportunity to clarify how we addressed it. To explore the potential sources of heterogeneity, we conducted a multivariable meta-regression analyses based on population characteristics, study design, geographical location (continent), number of participants, follow-up years, insomnia diagnostic criteria and diagnostic criteria for dementia. The findings from these analyses were as follows:

a. **Multivariable Meta-Regression:**

A Multivariate meta-regression analysis was performed to explore the potential sources of heterogeneity in the relationship between insomnia and dementia risk. The model included the following covariates: continent, follow-up duration, number of participants, study type, insomnia diagnosis criteria, and dementia diagnosis criteria. The results showed that no covariates was significantly associated with the effect size, including continent (*p*=0.924), follow-up years (*p*=0.524), number of participants (*p*=0.984), study type (*p*=0.527), and insomnia diagnosis criteria(*p*=0.621), and dementia diagnosis criteria (*p*=0.623), did not reach statistical significance. The details of meta-regression results with the risk of All-cause dementia are depicted in Table 3.

b. **Heterogeneity Impact:**

Despite these efforts, heterogeneity remained unexplained. This suggests that other variables that we did not measure may also contribute to the observed heterogeneity.

Through an extensive review of the literature, we identified that heterogeneity in meta-analyses generally arises from three sources: **clinical heterogeneity, methodological heterogeneity, and statistical heterogeneity[1].** Statistical heterogeneity is a result of clinical or methodological heterogeneity or the combination of two heterogeneities among the studies[1]. It is important to emphasize that clinical and methodological heterogeneity are **inevitable in any meta-analysis[2]**.

We have acknowledged this limitation in the revised manuscript and emphasized that the results should be interpreted with caution due to the high level of residual heterogeneity.

c. **Sensitivity Analyses:**

In addition to the meta-regression, we performed sensitivity analyses to test the robustness of our findings. Despite the persistence of heterogeneity, the results of the sensitivity analyses remained consistent, further supporting the reliability of our conclusions.

Based on these findings, we have expanded the discussion in the revised manuscript to provide a more detailed explanation of the meta-regression results and the role of insomnia diagnostic criteria in influencing heterogeneity. We hope these clarifications address your concerns, and we look forward to your further feedback.

**REFERENCE**

[1]Schulzke S. Assessing and exploring heterogeneity[M]//Principles and practice of systematic reviews and meta-analysis. Cham: Springer International Publishing, 2021: 33-41.

[2]Higgins JP. Commentary: Heterogeneity in meta-analysis should be expected and appropriately quantified. Int J Epidemiol. 2008;37(5):1158–60.

**Changes in the text:**

**Page 15-16, Line 360~368**: In the results section added:

## “Meta regression

A Multivariate meta-regression analysis was performed to explore the potential sources of heterogeneity in the relationship between insomnia and dementia risk. The model included the following covariates: continent, follow-up duration, number of participants, study type, insomnia diagnosis criteria, and dementia diagnosis criteria. The results showed that no covariates was significantly associated with the effect size, including continent (p=0.924), follow-up years (p=0.524), number of participants (p=0.984), study type (p=0.527), and insomnia diagnosis criteria(p=0.621), and dementia diagnosis criteria (p=0.623), did not reach statistical significance. The details of meta-regression results with the risk of All-cause dementia are depicted in **Table 3**.”

**Page 24-25, Line 515~533**: In the limitation and prospection section. Added:

“To our understanding, this systematic review represents the most extensive and thorough examination of dementia incidence among individuals with insomnia to date. We used a random-effects model to combine all effect sizes. Sensitivity analyses were performed to verify the robustness of the findings. We also used subgroup analyses and meta-regression to explore the sources of heterogeneity. Moreover, the generally high quality of the studies included in our review provides a level of confidence in the results. Nonetheless, our study does have limitations. Clinical heterogeneity and methodological heterogeneity due to differences in demographics and experimental designs are the main sources of statistical heterogeneity in this meta-analysis and cannot be completely avoided. Although we did not identify possible sources of heterogeneity, we speculate that a key limitation is the reliance on self-reported insomnia, which could lead to recall bias or misclassification. Additionally, the considerable heterogeneity observed suggests that other unmeasured factors could influence the outcomes. Therefore, the results of our meta-analysis should be interpreted with caution. Future research should prioritize the use of standardized diagnostic criteria for insomnia and incorporate objective sleep measures to reduce heterogeneity. Long-term follow-up studies, particularly those exceeding five years, are necessary to fully capture the chronic impact of insomnia on dementia risk. Additionally, large-scale, multi-center studies adjusting for key confounders will help identify at-risk populations and clarify the role of insomnia as a modifiable risk factor for dementia.”

**Comment 4:** Subgroup Analyses: The authors have conducted subgroup analyses based on continent, follow-up years, gender, and insomnia subtypes. While these analyses provide valuable insights, they also reveal substantial differences in the risk estimates across different subgroups. For example, the risk of dementia associated with insomnia is markedly higher in Asian populations compared to European and American populations. These differences may be due to a variety of factors, including genetic predisposition, cultural differences, or variations in healthcare systems. The manuscript would benefit from a more in-depth discussion of these potential explanations and their implications for the generalizability of the findings.

**Reply 4:** Thank you for your valuable feedback regarding the subgroup analyses. We appreciate your recognition of the insights provided by these analyses, and we acknowledge that the substantial differences in risk estimates across various subgroups, particularly between Asian and Western populations, merit further discussion.

To address this, we have expanded the discussion in the revised manuscript to provide a more in-depth exploration of the potential factors that could explain these variations, including:

Genetic Predisposition: A large case-control study [1] across multiple racial and ethnic groups confirms that the Apolipoprotein E4 (apoE4) genotype is a significant risk factor for late-onset AD, with its risk varying by race, sex, and ancestry. Specifically, the risk associated with apoE4 was higher in East Asians (OR, 4.54) than White (OR, 3.46). These findings emphasize that differently apoE4 status across populations, potentially explaining why different racial and ethnic groups show varying risks of AD.

Cultural Differences: Culture and healthcare systems play a key role in cognitive ability and dementia incidence. East Asian cultures exhibit notable differences in their perceptions of sleep compared to Western cultures. For instance, a study[2] comparing Japanese and European Canadian participants found that the Japanese group perceived a weaker connection between sleep and physical health. Additionally, they reported a significantly shorter ideal sleep duration. These cultural attitudes toward sleep may influence the frequency and severity of cognitive decline associated with insomnia, as reduced sleep duration and a lack of emphasis on its health benefits could exacerbate the long-term impact of sleep disorders.

Healthcare System Variations: Access to dementia care also differs greatly. In low-to-middle income countries of Asia, dementia is often under-recognized, and healthcare systems are not equipped to address it efficiently [3]. This contrasts with European countries that have more robust healthcare systems, where dementia is recognized earlier, and patients have better access to preventive care for conditions like insomnia[4]. This cultural and healthcare systems context may partly explain regional differences in how insomnia affects the risk of All-Cause dementia.

We have now included a more detailed discussion of these potential explanations in the revised manuscript and have also considered how these differences might affect the generalizability of our findings across populations.

We hope this additional context addresses your concern, and we look forward to your further feedback.

**REFERENCE**

1.	Belloy ME, Andrews SJ, Le Guen Y, Cuccaro M, Farrer LA, Napolioni V, et al. APOE genotype and Alzheimer disease risk across age, sex, and population ancestry. JAMA neurology. 2023;80(12):1284-94.

2.	Cheung BY, Takemura K, Ou C, Gale A, Heine SJ. Considering cross-cultural differences in sleep duration between Japanese and Canadian university students. PLoS One. 2021;16(4):e0250671.

3.	Ferri CP, Jacob K. Dementia in low-income and middle-income countries: different realities mandate tailored solutions. PLoS medicine. 2017;14(3):e1002271.

4.	Schmachtenberg T, Monsees J, Thyrian JR. Structures for the care of people with dementia: a European comparison. BMC Health Services Research. 2022;22(1):1372.

**Changes in the text:**

**Page 21-22, Line 467~488**: In the Subgroup Analyses Findings section. Added:

“We hypothesize that this phenomenon may be related to genetic predisposition, cultural differences, or variations in healthcare systems. A large case-control study[52] across multiple racial and ethnic groups confirms that the Apolipoprotein E4 (apoE4) genotype is a significant risk factor for late-onset AD, with its risk varying by race, sex, and ancestry. Specifically, the risk associated with apoE4 was higher in East Asians (OR, 4.54) than White (OR, 3.46). These findings emphasize that differently apoE4 status across populations, potentially explaining why different racial and ethnic groups show varying risks of AD. Culture and healthcare systems play a key role in cognitive ability and dementia incidence. East Asian cultures exhibit notable differences in their perceptions of sleep compared to Western cultures. For instance, a study [53] comparing Japanese and European Canadian participants found that the Japanese group perceived a weaker connection between sleep and physical health. Additionally, they reported a significantly shorter ideal sleep duration. These cultural attitudes toward sleep may influence the frequency and severity of cognitive decline associated with insomnia, as reduced sleep duration and a lack of emphasis on its health benefits could exacerbate the long-term impact of sleep disorders. Access to dementia care also differs greatly. In low-to-middle income countries of Asia, dementia is often under-recognized, and healthcare systems are not equipped to address it efficiently [54]. This contrasts with European countries that have more robust healthcare systems, where dementia is recognized earlier, and patients have better access to preventive care for conditions like insomnia [55]. This cultural and healthcare systems context may partly explain regional differences in how insomnia affects the risk of All-Cause dementia.”

**Comment 5:** Statistical Analysis: The statistical analysis in the manuscript appears to be performed appropriately and rigorously. The authors have used both fixed-effects and random-effects models to account for heterogeneity, and they have conducted sensitivity analyses to validate the robustness of their findings. The use of Egger’s test and Begg’s test to evaluate publication bias is appropriate, and the results suggest that publication bias is not a major concern in this meta-analysis.

**Reply 5:** Thank you for your positive feedback on the statistical analysis in our manuscript. We are pleased that you found our use of fixed-effects and random-effects models, as well as the sensitivity analyses, to be rigorous and appropriate for addressing heterogeneity. Additionally, we appreciate your recognition of the use of Egger’s test and Begg’s test to evaluate publication bias, and we are glad that these tests indicated that publication bias is not a major concern in this meta-analysis.

We are committed to maintaining the robustness and reliability of our findings, and we welcome any further suggestions or questions you may have regarding the statistical methods.

**Comment 6:** Interpretation of Findings: The interpretation of the findings is generally sound, and the authors have provided a thorough discussion of the potential mechanisms underlying the association between insomnia and dementia. The discussion of amyloid-beta (Aβ) deposition, Tau protein abnormalities, and the role of inflammation in the pathogenesis of dementia is well-supported by the existing literature. The manuscript also highlights the complex bidirectional relationship between sleep disorders and neurodegeneration, which is an important consideration.

**Reply 6:** Thank you for your positive feedback on the interpretation of our findings. We are pleased that you found our discussion of the potential mechanisms, including amyloid-beta (Aβ) deposition, Tau protein abnormalities, and the role of inflammation in dementia pathogenesis, to be well-supported by the existing literature.

We appreciate your recognition of our emphasis on the complex bidirectional relationship between sleep disorders and neurodegeneration, as this is a key aspect of our study. We have aimed to provide a thorough and balanced discussion, integrating these mechanisms to offer a deeper understanding of the link between insomnia and dementia.

**Comment 7:** However, the manuscript could be strengthened by a more detailed discussion of the limitations of the study and the potential impact of confounding factors. For example, many of the included studies relied on self-reported questionnaires to diagnose insomnia, which may introduce recall bias or misclassification. Additionally, while the authors have adjusted for several confounders, there may be residual confounding that could influence the results. A more explicit acknowledgment of these limitations would add transparency to the manuscript and provide a more balanced interpretation of the findings.

**Reply 7:** Thank you for your insightful comments regarding the limitations of the study and the potential impact of confounding factors. We fully agree that a more detailed discussion of these issues would enhance the transparency and balance of the manuscript. In response, we have revised the manuscript to address the following points:

**Self-reported questionnaires and recall bias:** As you rightly pointed out, many of the included studies relied on self-reported questionnaires to diagnose insomnia, which could introduce recall bias or misclassification. In the revised manuscript, we now explicitly acknowledge this limitation and discuss its potential impact on our findings. Specifically, self-reported insomnia may not always accurately reflect the true presence or severity of the condition, potentially leading to either underestimation or overestimation of the association between insomnia and dementia. To address this concern, we conducted a Multivariate meta-regression analysis to explore the potential sources of heterogeneity in the relationship between insomnia and the risk of dementia. The model included the following covariates: continent, follow-up duration, number of participants, study type, insomnia diagnosis criteria, and dementia diagnosis criteria. The results showed that no covariates was significantly associated with the effect size, including continent (*p*=0.924), follow-up years (*p*=0.524), number of participants (p=0.984), study type (*p*=0.527), and insomnia diagnosis criteria(*p*=0.621), and dementia diagnosis criteria (*p*=0.623), did not reach statistical significance. The details of meta-regression results with the risk of All-cause dementia are depicted in Table 3.

**Residual confounding:** we have adjusted for several known confounders in our analysis, the potential for residual confounding remains. While our meta-regression analysis did not identify any significant additional contributors to heterogeneity including the diagnostic criteria for insomnia and follow-up years, we acknowledge that certain confounding factors may not have been fully captured or controlled for in the included studies. Therefore, we have incorporated a more cautious interpretation of the results, recognizing that residual confounding cannot be entirely ruled out.

By incorporating these discussions into the revised manuscript, we aim to provide a clearer and more comprehensive understanding of the study’s limitations, particularly the potential impact of diagnostic bias and residual confounding. We appreciate your suggestion and believe these revisions add further transparency and rigor to the manuscript.

**Changes in the text:**

**Page 15-16, Line 360~368**: In the meta regression section. Added:

**“**A Multivariate meta-regression analysis was performed to explore the potential sources of heterogeneity in the relationship between insomnia and dementia risk. The model included the following covariates: continent, follow-up duration, number of participants, study type, insomnia diagnosis criteria, and dementia diagnosis criteria. The results showed that no covariates was significantly associated with the effect size, including continent (*p*=0.924), follow-up years (*p*=0.524), number of participants (p=0.984), study type (*p*=0.527), and insomnia diagnosis criteria(*p*=0.621), and dementia diagnosis criteria (*p*=0.623), did not reach statistical significance. The details of meta-regression results with the risk of All-cause dementia are depicted in **Table 3**.**”**

**Page 24, Line 516~519**: In the Limitations and Prospection section. Added:

“We used a random-effects model to combine all effect sizes. Sensitivity analyses were performed to verify the robustness of the findings. We also used subgroup analyses and meta-regression to explore the sources of heterogeneity.”

**Page 24-25, Line 521~533**: In the Limitations and Prospection section. Added:

**“**Clinical heterogeneity and methodological heterogeneity due to differences in demographics and experimental designs are the main sources of statistical heterogeneity in this meta-analysis and cannot be completely avoided. Although we did not identify possible sources of heterogeneity, we speculate that a key limitation is the reliance on self-reported insomnia, which could lead to recall bias or misclassification. Additionally, the considerable heterogeneity observed suggests that other unmeasured factors could influence the outcomes. Therefore, the results of our meta-analysis should be interpreted with caution. Future research should prioritize the use of standardized diagnostic criteria for insomnia and incorporate objective sleep measures to reduce heterogeneity. Long-term follow-up studies, particularly those exceeding five years, are necessary to fully capture the chronic impact of insomnia on dementia risk. Additionally, large-scale, multi-center studies adjusting for key confounders will help identify at-risk populations and clarify the role of insomnia as a modifiable risk factor for dementia.**”**

**Comment 8:** Ethical Considerations: The manuscript includes an ethics statement indicating that the data were analyzed anonymously and that the datasets used during the study are available from public databases. This is consistent with ethical guidelines for meta-analyses, and no major ethical concerns are noted.

**Reply 8:** Thank you for your positive feedback regarding the ethical considerations in our manuscript. We are pleased that the ethics statement and the use of publicly available, anonymized datasets were found to be in line with ethical guidelines for meta-analyses.

We are committed to adhering to the highest ethical standards, and we ensure that all data used in this study were obtained from publicly accessible databases, analyzed anonymously, and handled in accordance with ethical guidelines.

We appreciate your thorough review and are happy to address any further concerns if needed.

**Comment 9:** The manuscript could benefit from a more detailed explanation of the search strategy, including the specific keywords and search terms used, as well as any limitations in the search process. This would allow readers to better assess the comprehensiveness of the literature search.
**Reply 9:** Thank you for your valuable suggestions regarding the search strategy and the abstract.

Search Strategy: We appreciate your feedback on the need for a more detailed explanation of the search strategy. In the revised manuscript, we have expanded this section to include a comprehensive description of the specific keywords, search terms, and Boolean operators used across all databases. These improvements should help readers better assess the comprehensiveness of our literature search.

Boolean operators and truncation symbols are the usual knowledge of the search literature, and we have not yet reached the original text. Detailed explanation below:

**1. Boolean Operators:** Boolean Operators: Boolean Operators are used to search for a number of different combinations of keys, and to help us further control the search results. Commonly used keywords include: AND: Include both or multiple keywords at the same time in the search result. For example, the search for "Insomnia	AND Dementia" also includes research on "Insomnia" (insomnia) and "Dementia" (dementia).

OR：Use of the search, the result of the search is the least inclusive one. For example, "Insomnia OR Sleep disorders" includes research on "Insomnia" or "Sleep disorders".

NOT: Usage excludes inclusion of certain specific terms as a result. For example, "Dementia NOT Alzheimer" is not included in the study of dementia.

**2. Truncation Symbols:** Truncation Symbols that can be used as alternate symbols for truncated symbols; The most commonly used cut-off symbol is the star sign (*), which means that you can read it and have a specific translation.

Example: Search for "sleep" * General translation of "sleep", "sleeping", "sleepless", etc. Possessed by "sleep". This method is very useful when searching for a large number of different languages, which will save you time, especially if you are unsure of the completeness of the translation or format.

Combination use: While searching for existing numbers, the combination of cloth calculation marks and cut codes is always used. For example, "Insomnia OR Sleep* AND Dementia".

**Changes in the text:**

**Page 6, Line 198~209**: added:

“We utilized a combination of medical subject headings (MeSH) and free-text keywords to ensure comprehensive coverage of relevant studies. The search terms included combinations of ("Insomnia" OR "Sleep initiation and Maintenance disorders" OR "Early morning awakening") and ("Dementia" OR "Alzheimer Disease" OR "Vascular Dementia") along with ("Risk"). Additionally, Boolean operators were applied to refine the results, and truncation symbols were used where applicable to capture variant word endings. The search was not limited by language or publication year, allowing us to capture studies from diverse geographical regions and timeframes. Furthermore, to maximize the comprehensiveness of the search, we reviewed reference lists of previous meta-analyses and systematic reviews [12-15], identifying any additional studies that may meet our inclusion criteria. Detailed search strategies, including the exact terms and filters used, are provided in **Supplementary Tables S1-S3**.”

**Comment 10:** The authors have provided a clear and concise abstract, but it would be helpful to include a brief mention of the limitations of the study in the abstract as well, to provide a more balanced summary of the findings.

**Reply 10:** We also acknowledge your suggestion to include a brief mention of the study's limitations in the abstract. In the revised version, we have added a concise statement highlighting the key limitations of our study, including the heterogeneity of the included studies and potential publication bias. This will provide a more balanced summary of our findings and ensure that readers are aware of the limitations upfront.

We hope these revisions address your concerns, and we appreciate your thorough review. We look forward to your further feedback.

**Changes in the text:**

**Page 3, Line 152~155**:

“Insomnia could be a risk factor for all-cause dementia. Subgroup analysis of the literature from different continents, follow-up times, and sexes also confirms that insomnia was more likely to occur as all-cause dementia. ”

Change to:

“These findings suggest that insomnia may significantly contribute to the risk of all-cause dementia, highlighting the importance of early intervention and management of insomnia. Despite our efforts to minimize and explore the sources of heterogeneity, it still remained, and therefore our results should be interpreted with caution.”

**Comment 11:** The manuscript is generally well-written, but there are a few minor grammatical errors and typos that should be corrected in the final version.

**Reply 11:** We sincerely appreciate your insightful feedback on our manuscript. Thank you for pointing out the grammatical errors and typos. We have corrected these grammatical and spelling errors in the revised manuscript to deliver a polished and clear manuscript.

Your detailed review has been extremely helpful, and we look forward to any further suggestions you might have.

**Changes in the text:**

**Page 4, Line 164~165**:

Currently, many interventions are attempted to treatment, but the effects are still unsatisfactory

Change to:

“Currently, many interventions are attempted for treatment, but the effects are still unsatisfactory.”

**Page 5, Line 179~180**:

Previous meta-analysis [12-15] have showen the relationship between insomnia and risk of all-cause dementia, encompassing AD and VD.

Change to:

**“**Previous meta-analysis [12-15] have shown the relationship between insomnia and the risk of all-cause dementia, encompassing AD and VD.**”**

**Page 5, Line 183~184**:

“Current hypotheses suggest potential associations with neuronal inflammation [19], disrupted amyloid-β protein metabolism [20], and cerebrospinal fluid Tau protein level [21].”

Change to

“Current hypotheses suggest potential associations with neuronal inflammation [19], disrupted amyloid-beta (Aβ) protein metabolism [20], and cerebrospinal fluid Tau protein level [21].”

**Comment 12:** In conclusion, the manuscript presents a valuable contribution to the literature on the relationship between insomnia and dementia risk. The findings are supported by a rigorous and systematic approach to data synthesis, although the significant heterogeneity observed in some analyses warrants caution in the interpretation of the results. With some revisions, the manuscript has the potential to make a meaningful impact on the understanding of sleep disorders and neurodegenerative diseases.

**Reply 12:** Thank you for your encouraging and positive feedback on our manuscript. We are truly grateful for your recognition of the value that our study brings to the understanding of the relationship between insomnia and dementia risk.

Regarding the significant heterogeneity observed in some of our analyses, we fully acknowledge the importance of careful interpretation. As noted in the manuscript, we have taken multiple steps to address this issue, including conducting subgroup analyses, meta-regression, and sensitivity analyses, as well as providing a cautious interpretation of the findings. We have also revised the manuscript to further clarify these points and ensure transparency in our approach.

Your constructive comments have been incredibly helpful, and we believe the revisions have strengthened the manuscript. We are excited about the potential impact of this work and appreciate your invaluable input in helping us refine it.

**Comment 13:** I recommend that the authors address the concerns regarding heterogeneity and provide a more detailed discussion of the study's limitations. With these revisions, the manuscript would be suitable for publication.

**Reply 13:** Thank you for your constructive feedback and for recognizing the potential of our manuscript. We appreciate your recommendation to address the concerns regarding heterogeneity and provide a more detailed discussion of the study’s limitations.

In response your concerns, we have expanded the discussion in the revised manuscript to offer a more comprehensive explanation of the heterogeneity observed, including the results of our subgroup analyses, meta-regression, and sensitivity analyses. Additionally, we have provided a more detailed discussion of the study’s limitations, including the impact of self-reported data, potential residual confounding, and other factors that may influence the interpretation of our findings.

We are confident that these revisions have strengthened the manuscript, and we are grateful for your thoughtful suggestions that have helped improve the clarity and transparency of our work.

Thank you again for your recommendation, and we look forward to your further feedback.

**Comment 14:** Response to the content of the reviewer's comments attachment.

**Reply 14:** We are truly humbled and deeply grateful for the extraordinary effort you have put into reviewing our manuscript. Your meticulous, line-by-line feedback, spanning six pages, has not only provided us with invaluable insights but also demonstrated a level of dedication and care that goes far beyond our expectations. We sincerely appreciate the time and energy you invested in guiding us through each step of the revision process.

Thanks to your thorough and thoughtful suggestions, we have implemented every one of your recommended changes. We genuinely believe that your detailed feedback has significantly elevated the quality of our manuscript. This experience has been incredibly enriching, and we are confident that our work is now much stronger because of your invaluable contributions.

Once again, we extend our heartfelt thanks for your exceptional guidance and for being such an engaged and supportive reviewer. Your input has made an enormous difference, and we feel privileged to have had the opportunity to benefit from your expertise.

We really appreciate your positive and insightful suggestions on our manuscript. Under your kind help and professional guidance, we believe that our manuscript has been improved substantially. We are looking forward to your further suggestions.

Sincerely yours,

Xiaoming Shen, Ph.D, M.D, Prof.

Email: sxmdoc@hactcm.edu.cn
